# Molecular insights into silver nanoparticle resistance in *Acinetobacter baumannii* and unique adaptations to ionic silver

**DOI:** 10.1038/s44259-025-00161-9

**Published:** 2025-11-25

**Authors:** Oliver McNeilly, Daniel G. Mediati, Matthew J. Pittorino, Riti Mann, Bill Söderström, Mehrad Hamidian, Cindy Gunawan

**Affiliations:** 1 Australian Institute for Microbiology and Infection, University of Technology Sydney, Ultimo, NSW Australia; 2 Department of Infection Biology, London School of Hygiene and Tropical Medicine, London, UK

**Keywords:** Experimental evolution, Bacterial evolution, Antimicrobial resistance, Microbiology, Biofilms, Gene regulation

## Abstract

This research provides molecular insights into the evolutionarily adapted defense mechanisms of a bacterial pathogen against the complex antimicrobial activity of silver nanoparticles at the transcriptional level. The Gram-negative, biofilm-forming bacterium *Acinetobacter baumannii* upregulated outer membrane proteins along with genes involved in membrane and capsule synthesis, suggestive of enhanced cell surface defense. An increase in surface-attached biofilm colonies in nanosilver-resistant *A. baumannii* (NAg^R^) appeared to be associated with enhanced cell membrane integrity and a greater production of extracellular polymeric substances (EPS), the matrix that protects the resident colony. In response to reactive oxygen species (ROS), a recognized toxicity characteristic of the nanoparticle, NAg^R^ upregulated its oxidative stress management system, specifically involving ROS scavenger enzymes and opportunistic metal efflux pumps. Majority of these modified defense mechanisms manifested in the resistant bacterium, while absent in the wild-type strain. This study also details the unique defenses of an ionic silver-tolerant *A. baumannii* (Ag^T^) variant, which evolved from the same parental strain as NAg^R^. Despite similarities in cell surface and biofilm defense traits, the slower-to-kill tolerant strain exclusively upregulated multidrug efflux systems and respiratory chain enzymes, thought to maintain enhanced respiratory activity, a reported tolerance characteristic. While these findings would benefit from further validating experimental work, identification of these stable defense mechanisms can help better elucidate the complex nature of bacterial NAg adaptation phenomena.

## Introduction

The medical and commercial indiscriminate use of antimicrobial agents, along with diminishing pharmaceutical investment toward novel antibiotic development, have been the primary drivers of global antimicrobial resistance (AMR)^1–3^. Between 2019 and 2021, systematic analyzes estimated over 4 million deaths were attributable to AMR globally^4^. One of the major contributors to the AMR crisis is the opportunistic Gram-negative coccobacillus bacterium *Acinetobacter baumannii*. Carbapenem-resistant *A. baumannii* has been classified as the number one critical level priority pathogen by the World Health Organization since 2017^5,6^. This species is among the leading cause of nosocomial (hospital-acquired) infection, with for instance, 80–97% of reported cases in Southern Europe, the Middle East, and North Africa, due to multidrug-resistant (MDR) strains^7,8^. Global mortality rates from MDR *A. baumannii* have ranged from 24 to 83%, with higher incidences occurring in elderly and/or immunocompromised individuals^9–11^. Treatment options for MDR (including carbapenem-resistant) *A. baumannii* infections are currently limited to antibiotics such as polymyxins (i.e., colistin), and yet, resistance to these last-line treatments has been identified globally^12–14^.

The growing threat of AMR calls for immediate and innovative solutions. Nanotechnology has gained significant traction in the biomedical sector in recent years^15^. Silver nanoparticles (NAg) are now one of the most commercialized products of nanotechnology, due to their unique physicochemical characteristics and broad-spectrum antimicrobial properties. The nanoparticles have been shown to be equally effective against MDR pathogens, including MDR *A. baumannii*^16–19^. NAg is currently a major alternative antimicrobial and has been incorporated in medical devices—such as in wound dressings and catheters—and increasingly in consumer products, including household appliances, with the primary aim of controlling pathogen growth^20–22^. NAg target microbes through the cell-killing activities of the soluble silver that leach from the nanoparticles, including ionic silver (Ag^+^), and the solid silver particulates that remain after leaching^23–25^. The antimicrobial activity of silver has been largely associated with the generation of cellular reactive oxygen species (ROS)^24,26^. Among other potential ROS-generation pathways, studies have indicated that both NAg and leached Ag⁺ can target membrane-bound respiratory chain components in bacteria^24,27^. The disruption of the electron transport chain causes premature leakage of electrons, thereby reducing molecular oxygen (O_2_), present in the cytoplasm, into superoxide radicals (O_2_^•-^)^24^. Studies have further suggested that O_2_^•-^ radicals (and leached Ag^+^) target iron-sulfur (Fe-S) clusters that are present in many important enzymes and proteins^28,29^. Notably, the intracellular presence of leached Ag^+^ has also been linked to potential Trojan-horse type toxicity of NAg, with intracellular ion leaching following particle uptake^30^. The targeting of Fe-S clusters could consequently release Fenton-active Fe(II) ions, which can react with cellular H_2_O_2_ to generate highly reactive hydroxyl radicals (^•^OH)^24,29^. Hydroxyl and superoxide radicals are known to inactivate proteins, for instance, by oxidizing the sulfur atom in the amino acid methionine to sulfoxide and/or oxidizing the thiol group (-SH) in cysteine to thiyl radical (RS^•^)^29,31^. In DNA, hydroxyl radicals target H-bonds present in the nucleotide base-pairs, as well as in the sugar moieties of the sugar-phosphate backbone, causing nucleotide cleavage^32^. The thiyl radical is also recognized for its lipid peroxidation activities, particularly targeting membrane phospholipids^33^.

The increasing and often indiscriminate use of NAg has raised concerns over the development of silver-adapted pathogens, despite the complex, multi-targeting antimicrobial toxicity of silver. Resistance to ionic silver has been reported in bacteria for decades, including in *A. baumannii*^34–38^. These resistance traits can arise endogenously, such as through mutation, or be acquired exogenously via the uptake of mobile genetic elements, including plasmids carrying *sil* genes. In the latter case, studies have identified the presence of plasmids harboring the nine-gene silver efflux cluster *silCFBA*(ORF105aa)*PRSE* in *A. baumannii*^39^. Evidence of nanosilver resistance was first reported in 2013 in an environmental bacterium following prolonged exposure^40^. Indeed, such occurrences of nanosilver adaptation have been increasingly observed in both Gram-negative and Gram-positive bacterial species, including clinically-relevant strains^39,41–44^. Recently, we reported the first known evidence of evolutionarily-acquired nanosilver resistance in *A. baumannii* (model strain ATCC 19606), following long-term exposure to the nanoparticle^45^. With no prevalence of any *sil* genes, neither in its chromosome nor in native plasmids, the bacterium was able to evolve harder-to-kill resistance traits, which resulted in up to fivefold increase in nanosilver MIC (minimum inhibitory concentration) of that of the wild-type strain. The adaptation characteristics still manifested even after growth in antimicrobial-free conditions and were associated with the development of stable single-nucleotide polymorphisms^45^. Rationally, observation of these stable mechanisms prompted further in-depth work to identify these evolved defense characteristics at the molecular level.

In this study, we investigated changes in gene expression and observable phenotypic traits of nanosilver-resistant *A. baumannii* (herein referred to as NAg^R^ or resistant strain) in response to sub-lethal and lethal nanosilver concentrations. When compared to the wild-type (WT), the resistant strain exhibited both inherent and adaptive defense mechanisms, which we define herein as “primary” and “secondary” defenses, respectively. We refer to primary defenses as intrinsic mechanisms observable in the WT that are upregulated at higher expression levels in the resistant strain (NAg^R^), while secondary defenses refer to novel adaptation mechanisms exclusive to the resistant strain. In addition to the expected defense mechanisms that mitigate ROS-associated toxicity of nanosilver, our study revealed stable cell surface defense mechanisms and modified biofilm growth behavior in the bacterium. Furthermore, we examined the gene expression and phenotypic profiles of an ionic silver-tolerant *A. baumannii* strain (herein referred to as Ag^T^ or tolerant strain), which was evolved from the same ATCC 19606 parental strain via simultaneous (30-day) long-term exposure to silver cations^45^. Despite sharing several silver-relevant defense traits, Ag^T^ is physiologically distinct from NAg^R^, having evolved unique “Ag^+^-specific” mechanisms. Notably, nearly all mutated genes identified in the silver-adapted strains from our previous work showed no expression changes following the transcriptomic analysis herein, suggesting that the defense mechanisms involve more complex regulatory and physiological adaptations beyond mutation. Overall, these multi-layered findings provide valuable insights into the molecular basis of silver resistance and could help highlight potential molecular targets to counteract evolutionary adaptation.

## Results and discussion

### Cell surface defense and physiological changes

**Fig. 1:**
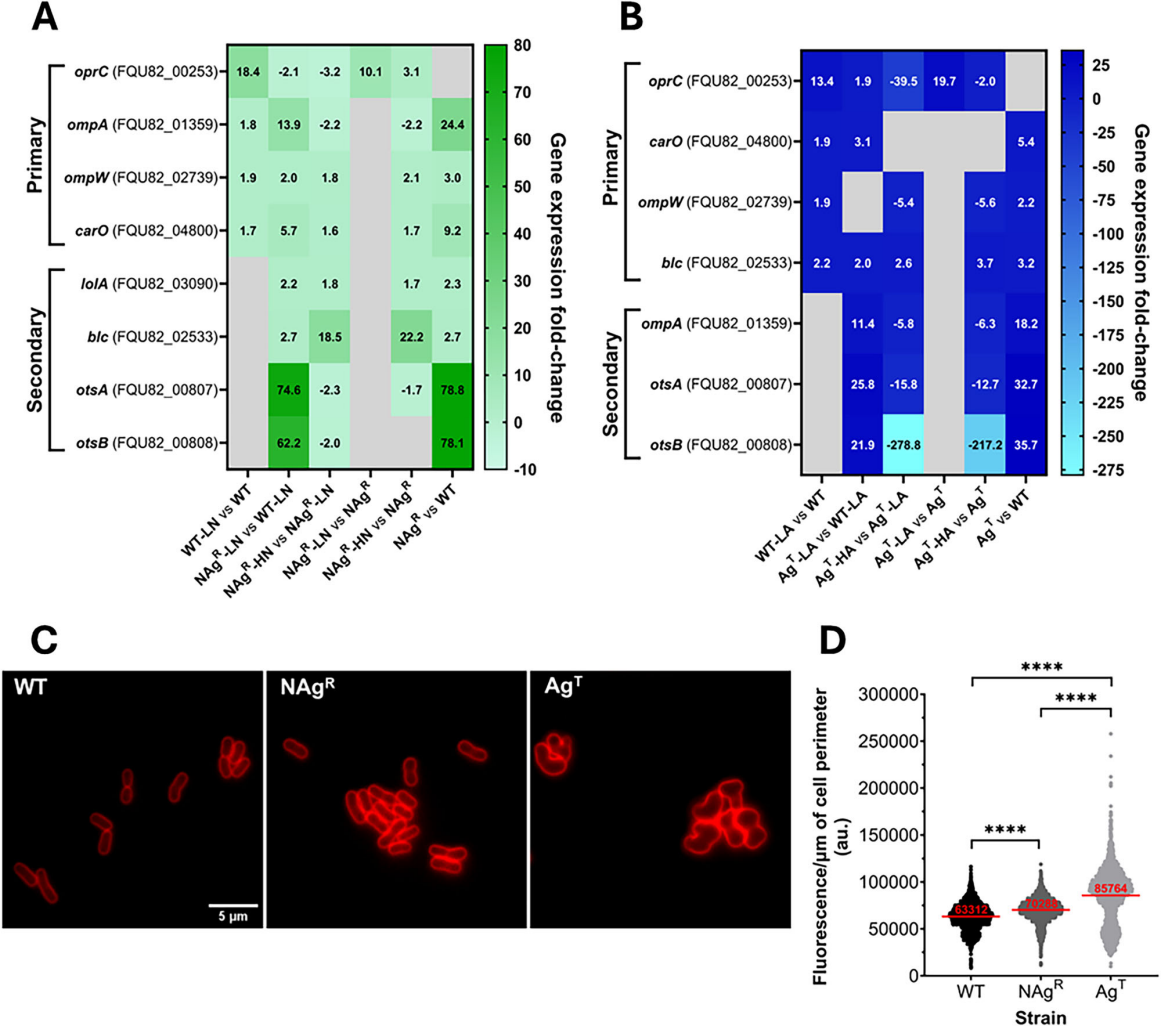
Cell surface defense mechanisms identified in silver-adapted *A. baumannii.* **A**, **B** Comparative analysis of chromosomal mRNA transcript levels of outer membrane protein (*oprC*, *ompA*, *ompW*, *carO*), membrane synthesis (*lolA*, *blc*) and capsule synthesis (*otsA*, *otsB*) genes in the wild-type (WT), nanosilver-resistant (NAg^R^) and ionic silver-tolerant (Ag^T^) strains, upon exposure to low silver concentrations (LN = ‘low’ nanosilver; 0.5 µg Ag/mL [0.5 × MIC], LA = ‘low’ ionic silver; 1 µg Ag/mL [0.5 × MIC] and high silver concentrations (HN = ‘high’ nanosilver; 3 µg Ag/mL [3 × MIC], HA = ‘high’ ionic silver; 3 µg Ag/mL [3 × MIC]). The differentially expressed genes (log_2_-fold change cut-off threshold ≥0.58 (≥1.5-fold change), *p*-adjusted ≤0.01, grey cells indicate statistically insignificant *p*-adj >0.01 changes) were captured at 30 min of silver exposures in the exponential growth phase. Also shown are the physiological gene expression changes when in the absence of silver. **C** Phenotypic studies of bacterial membrane with lipophilic fluorescence staining of WT and the silver-adapted strains (scale bar = 5 µm) at 30 min growth in the exponential phase. **D** Quantitative analysis of fluorescence intensity per µm of cell perimeter (au.). Each data point represents a single cell measurement, with the horizontal bar showing the mean fluorescence from 2000 to 4000 cells analyzed, per strain. Statistical analysis (unpaired *t*-test with Welch’s correction, to account for unequal variances and large data sets) showed statistically significant differences in fluorescence intensity between each strain (****p < 0.0001). The mRNA and phenotypic work were performed in a minimum of three biological replicates (independent bacterial colony isolates).

Here, we first describe the primary defense mechanism employed by *A. baumannii* against nanosilver antimicrobial targeting, with a focus on transcriptomic and phenotypic changes in cell envelope components that evolved through long-term NAg exposure. The wild-type (WT) strain showed increased expression changes in genes that encode outer membrane-embedded proteins upon exposure to sub-lethal NAg (0.5 × MIC, 0.5 µg Ag/mL, referred to as ‘low’ nanoparticle concentration). Firstly, *oprC*, encoding the TonB-dependent copper channel OprC, was upregulated by ~18-fold, relative to the cell-only (i.e., silver-free or untreated) control samples (Fig. 1A, *p*-adjusted ≤0.01). Increased OprC expression in *A. baumannii* has been indicated to enhance sequestration of toxic levels of Cu^+^ within the periplasmic space. Protein structure-based studies further suggest that OprC plays a role in trapping silver particulates and leached Ag^+^ (like Cu^+^ - a soft acid) in the periplasm^46–50^. The NAg exposures also caused the WT strain to upregulate outer membrane proteins which are highly conserved in *A. baumannii* lineages, with ~1.7–1.9-fold upregulations of *ompA*, *ompW* and *carO*, relative to the cell-only samples. OmpA is the most conserved outer membrane protein in *A. baumannii*, with studies linking its upregulation to enhance membrane integrity, and in turn, higher resilience against many surface-targeting antimicrobial agents^51–55^. The outer membrane protein OmpW has also been associated with bacterial defense through improved cell membrane integrity^56^. The porin CarO is also highly conserved in *A. baumannii*^57^. Upregulation of this porin has been suggested to enhance cell-to-surface attachment and promote biofilm growth^51,58^. Indeed, studies have also linked OmpA and OmpW to biofilm formation in *A. baumannii*, which, as later shown in this work, could contribute to NAg defense^51,57,59,60^. Upregulation of these outer membrane proteins was also observed with ionic silver exposure (Ag^+^, supplied as AgNO_3_). A ~1.9–13-fold increase in expressions of *oprC*, *ompW* and *carO* were noted in the WT when exposed to the ‘low’ ionic silver concentration (0.5 × MIC, 1 µg Ag/mL), relative to the cell-only control (Fig. 1B).

These membrane proteins were notably more upregulated in the resistant strain. The resistant strain (NAg^R^) showed increased expressions of *ompA*, *ompW* and *carO* by ~2.0–14-fold, relative to the WT, when exposed to the low nanoparticle concentration (0.5 × MIC) (Fig. 1A). Exposure to ‘high’ nanoparticle concentration (3 × MIC, 3 µg Ag/mL) led to further ~1.6–1.8-fold upregulations of *ompW* and *carO* in the resistant strain, relative to the low concentration exposures (0.5 × MIC). Notably, *ompA* was downregulated by ~2.2-fold, following high concentration exposure, relative to the low concentration exposure. Comparable transcriptomic changes were also seen in the tolerant strain, with ~11 and ~3.1-fold increased expressions of *ompA* and *carO*, respectively, following exposure to the low ionic silver concentration (0.5 × MIC), relative to WT (Fig. 1B). Downregulation of *ompA*, by ~5.8-fold, was also observed in Ag^T^, at the high concentration exposure (3 µg Ag/mL), relative to the low concentration exposure. Downregulation of *ompA* has been previously reported in silver-exposed *Escherichia coli*, however, the exact reason for this is unclear^35^. Next, we observed downregulation of *oprC* in both NAg^R^ and Ag^T^ upon exposure to low (0.5 × MIC) and high (3 µg Ag/mL) silver concentrations. Specifically, *oprC* was downregulated by ~2.1 and ~3.2-fold in NAg^R^ relative to WT at the low and high concentrations, respectively, and by a more substantial ~40-fold in Ag^T^ at the high concentration compared to the low concentration exposure. The reasons for these downregulations are still not clear, although we predict they may relate to management of periplasmic silver levels^61^.

Next, we found that the resistant strain upregulated several lipoprotein carriers, which were not seen in the wild-type strain, hence defined as secondary defense mechanisms (Fig. 1A). Exposure to the high nanoparticle concentration (3 × MIC, 3 µg Ag/mL) resulted in ~1.7- and ~22-fold expression increases of *lolA* and *blc* (lipocalin), respectively, in the NAg^R^ strain, relative to the cell-only samples. No statistically significant upregulations were observed in NAg^R^ with the low concentration nanoparticle exposures (0.5 × MIC), relative to the cell-only samples, however, ~2.2 and ~2.7-fold respective upregulations were detected, relative to WT. LolA is a periplasmic chaperone that forms a part of the LolABCDE protein complex. LolA, like the outer membrane-embedded protein lipocalin, transports lipoproteins for outer membrane biogenesis^62,63^. LolA, along with the ATP-binding cassette transporter LolCDE, shuttles newly synthesized lipoproteins to the outer membrane^62^. Increased expressions of the Lol system and lipocalins have been observed in antimicrobial exposure cases in bacteria, which are thought to increase lipoprotein delivery to the outer membrane for repair^62,63^. Relevant to nanosilver toxicity, studies report that Gram-negative bacteria, such as *E. coli*, upregulate lipocalin synthesis in response to oxidative stress^64–66^. Upregulation of *blc* was also observed in Ag^T^, by ~3.7-fold with the high concentration exposures (3 µg Ag/mL), relative to the cell-only samples, and by ~2.0-fold with the low concentration exposures (0.5 × MIC), relative to WT (Fig. 1B).

The observed upregulation of *otsA* and *otsB* in the resistant strain—encoding trehalose-6-phosphate synthase and trehalose-6-phosphate phosphatase, respectively—is also considered a secondary defense mechanism herein^67,68^. Trehalose, a structural component of capsular polysaccharide (CPS), is known to accumulate in bacteria under stress conditions, supporting its role in adaptation and antimicrobial protection^69^. The NAg^R^ strain showed a significant increase in the expression of *otsA* and *otsB* upon exposure to the low nanoparticle concentration (0.5 × MIC), with fold changes of ~62 and ~75, respectively, relative to WT (Fig. 1A). In *A. baumannii*, increased trehalose synthesis has been associated with enhanced CPS density and thickness, which is thought to contribute to cell envelope protective effects^68^. In fact, upregulations of CPS synthesis genes have been previously observed with NAg exposures, in *E. coli* and *Staphylococcus aureus*^70,71^. Upregulations of *otsA* and *otsB* were also observed in Ag^T^, by ~26 and 22-fold, relative to WT, when exposed to the low ionic silver concentration (0.5 × MIC) (Fig. 1B). These genes, however, were downregulated at the high silver concentration exposures (3 µg Ag/mL, nanoparticle and ionic silver), by ~2.3 and 2.0-fold, respectively, in NAg^R^, and by the more substantial ~16 and ~279-fold, respectively, in Ag^T^, relative to the low silver concentration exposures (0.5 × MIC). The expression downregulations, particularly the extensive ones seen with Ag^T^, are thought to form part of the bacterium’s strategy to conserve energy for more silver-specific defense mechanisms that are uniquely manifested in this strain, as described later.

The transcriptomic changes in the resistant strain were, in fact, already manifested in the absence of silver. Upregulations of the outer membrane proteins (*ompA*, *ompW*, *carO*) by ~3.0–24-fold, the lipoprotein carriers (*lolA* and *blc*) by ~2.3 and 2.7-fold, respectively, and again, the notably high upregulations of the capsule synthesis enzymes (*otsA* and *otsb*) by ~78–79-fold, were observed in the NAg^R^ strain, relative to the WT strain (cell-only, Fig. 1A). These findings suggest that stable cell envelope integrity and membrane repair mechanisms have evolved in the resistant strain. Detailed fluorescence analysis of NAg^R^ with FM4-64 showed increased membrane fluorescence (per µm of the cell perimeter), when compared to WT (*p* < 0.0001, cell-only, Fig. 1C, D). The highly lipophilic dye (firstly) stains the bacterium outer membrane (before diffusing intracellularly), intercalating among membrane phospholipids and lipoproteins^72,73^. Consistent with transcriptomic data, the increased membrane fluorescence intensity may reflect greater membrane density, although definitive conclusions are limited by the properties and standard purpose of this dye. These phenotypic changes were indeed also observed with Ag^T^ with higher membrane fluorescence intensity, relative to WT (*p* < 0.0001, cell-only, Fig. 1C, D). Upregulations of *ompA*, *ompW*, *carO* by ~2.2–18-fold, *blc* by ~3.2-fold, and more substantially, *otsA* and *otsB* by ~33- and 36-fold, respectively, were detected in Ag^T^, relative to WT (cell-only, Fig. 1B). Despite the transcriptomic and phenotypic similarities, a more detailed analysis revealed different membrane fluorescence traits with Ag^T^ when compared to NAg^R^, which could associate with the unique morphological transformation observed with the former, as later described.

### Evolved changes in biofilm growth behavior

**Fig. 2:**
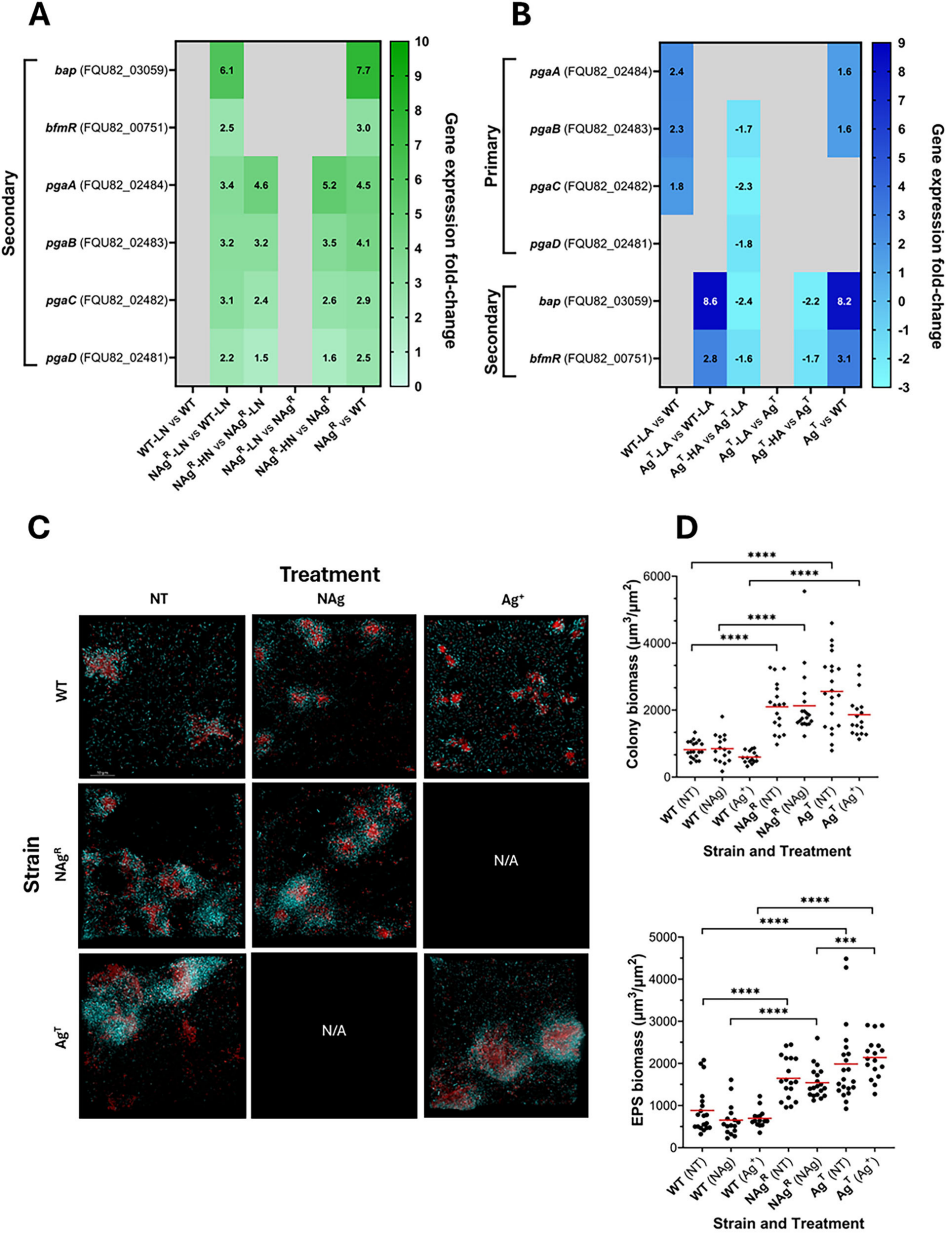
Changes in biofilm growth behavior in silver-adapted *A. baumannii.* **A**, **B** Comparative analysis of chromosomal mRNA transcript levels of cell-to-surface adherence associated genes (*bap*, *bfmR*) and EPS synthesis genes (*pgaABCD*) in WT, NAg^R^ and Ag^T^, upon exposure to low silver concentrations (LN = ‘low’ nanosilver; 0.5 µg Ag/mL [0.5 × MIC], LA = ‘low’ ionic silver; 1 µg Ag/mL [0.5 × MIC] and high silver concentrations (HN = ‘high’ nanosilver; 3 µg Ag/mL [3 × MIC], HA = ‘high’ ionic silver; 3 µg Ag/mL [3 × MIC]). The differentially expressed genes (log_2_-fold change cut-off threshold ≥0.58 (≥1.5-fold change), *p*-adjusted ≤0.01, grey cells indicate statistically insignificant *p*-adj >0.01 changes) were captured at 30 min of silver exposures in the exponential growth phase. Also shown are the physiological gene expression changes when in the absence of silver. **C** Fluorescent microscopy images of biofilms formed by WT and the silver-adapted strains when exposed to silver (0.5 × MIC of NAg and/or Ag^+^). Also shown are the respective cell-only (untreated) control samples. Cyan color showed surface-attached bacterial colonies, while red color showed EPS mass, scale bar = 10 µm. **D** Quantitative analysis revealed total colony (µm^3^/µm^2^) and EPS mass (µm^3^/µm^2^) of each strain under each respective silver exposures. Each data point represents calculated biomass surface coverage from individual images, with the horizontal bar showing the mean data from 15 to 20 images, per strain, per treatment. Statistical analysis (unpaired *t*-test with Welch’s correction) showed statistically significant differences in bacterial colony and EPS abundance between strains and treatments (***p < 0.001, ****p < 0.0001). The mRNA and phenotypic work were performed with a minimum of three biological replicates (single isolated colonies).

In line with the previously described upregulations of outer membrane proteins, as well as proteins involved in membrane and capsule synthesis, this study also revealed increased expression levels of other biofilm growth-associated genes that evolved in response to prolonged silver exposure. When bacterial communities grow as surface-attached biofilms, they are protected from antimicrobial targeting by a matrix of polysaccharides, proteins, lipids, and extracellular DNA, collectively known as the extracellular polymeric substance (EPS), which is produced by the residing bacteria^74–78^. NAg^R^ upregulated the *pgaABCD* operon by ~1.6–5.2-fold when exposed to the high NAg concentration (3 µg Ag/mL), relative to the cell-only samples (*p*-adj ≤0.01, Fig. 2A). At the low nanoparticle exposures (0.5 × MIC), ~2.2–3.4-fold upregulations were observed in NAg^R^, relative to WT. This operon encodes the membrane-bound protein complex PgaABCD, which synthesizes the exopolysaccharide poly-β-(1-6)-*N*-acetylglucosamine (PNAG), a major EPS constituent in *A. baumannii* biofilms^79^. The transcriptomic changes, however, were not seen in the WT strain upon silver exposure, relative to cell-only samples, and is hence defined as a secondary defense mechanism. Our phenotypic studies on biofilm growth supported the transcriptomic changes. As shown in Fig. 2C, D, the resistant strain was associated with not only a higher presence of surface-attached bacterial colonies, but also a greater extent of EPS formation. Upon exposures to nanosilver (0.5 × MIC), a ~2.4–2.5-fold more colonies (~2130 µm^3^/µm^2^) and EPS formation (~1545 µm^3^/µm^2^) were detected with the NAg^R^ biofilms, when compared to WT (~850 and ~654 µm^3^/µm^2^, respectively, *p* < 0.0001). The increase in EPS formation, as reported in earlier studies, was likely a result of increased *pgaABCD* operon upregulation seen with the resistant strain, relative to WT, conferring greater antimicrobial defense^77,80^. The (typically) net negatively-charged EPS has been indicated to adsorb and sequestrate positively-charged heavy metals, including silver^81,82^. The presence of more colonies with the resistant strain biofilms could associate with the earlier described upregulations of the cell-to-surface adherence-associated outer membrane *ompA*, *ompW* and *carO*, as well as the membrane synthesis *lolA*, *blc* and capsule synthesis *otsA*, *otsB*, observed with the strain, relative to WT (at 0.5 × MIC nanoparticle exposure, Fig. 1A), which is thought to enhance surface colonization capabilities. Further, the NAg^R^ strain was also found to upregulate *bap*, which encodes the Bap protein – one of the most conserved biofilm-associated proteins in *A. baumannii*. This protein is essential for both cell-to-surface adherence and biofilm maturation^83^. A ~6.1-fold increased *bap* expression was detected in NAg^R^, relative to WT, upon nanoparticle exposures (0.5 × MIC) (Fig. 2A). The resistant strain also upregulated the conserved regulatory gene *bfmR*, part of the BfmR/S transcriptional stress response regulatory system, which, again, has been previously linked to biofilm formation in *A. baumannii*^84,85^. A ~2.5-fold increase in *bfmR* expression was observed in NAg^R^, relative to WT, upon nanoparticle exposures (0.5 × MIC). Studies have in fact, reported BfmR/S transcriptional regulations of *bap*, *ompA*, as well as *otsA*, *otsB*^84,85^. The designation of enhanced biofilm growth as a secondary defense mechanism for nanosilver resistance is supported by the lack of observable increases in both gene expressions (*pgaABCD*, *bap*, *bfmR*) and phenotype (surface-attached bacterial colonies and EPS formation) in the WT strain when exposed to the nanoparticle (0.5 × MIC) compared to the cell-only samples. (Fig. 2A, C, D).

Further transcriptomic analysis of the resistant strain revealed upregulation of EPS-related genes and cell-to-surface adherence genes, even in the absence of silver. This suggests the presence of stable biofilm-associated defense mechanism(s), as supported by the phenotypic evidence. Upregulations of *pgaABCD* by ~2.5–4.5-fold and *bap* by ~7.7-fold were detected in NAg^R^, relative to WT (cell-only, Fig. 2A), and correspondingly, a ~1.9-fold higher extent of EPS formation (~1645 µm^3^/µm^2^) and ~2.6-fold more colony presence (~2100 µm^3^/µm^2^) was seen in NAg^R^ compared to WT (~885 and ~820 µm^3^/µm^2^, respectively, *p* < 0.0001, cell-only, Fig. 2C, D). Upregulation of the biofilm-associated stress response transcriptional regulator *bfmR* was also detected in the resistant strain, by ~3.0-fold, relative to WT (cell-only, Fig. 2A). To recall, NAg^R^ also upregulated the cell-to-surface adherence-associated genes *ompA*, *ompW*, *carO*, *lolA*, *blc*, *otsA*, *otsB*, with cell-only, relative to WT (Fig. 1A).

Enhanced biofilm growth characteristics were also seen in the tolerant strain both in the presence and absence of silver, the latter of which indicates it is a stable defense trait. Ag^T^ formed ~3.2-fold more EPS (~2205 µm^3^/µm^2^) with ~2.9-fold more presence of colony biomass (~1755 µm^3^/µm^2^) when exposed to ionic silver (0.5 × MIC), relative to WT (~700 and ~596 µm^3^/µm^2^, respectively, *p* < 0.0001, Fig. 2C, D). Without silver, a ~2.2-fold more EPS (~1990 µm^3^/µm^2^) and ~3.1-fold more colonies (~2560 µm^3^/µm^2^) were observed, relative to WT (~885 and ~820 µm^3^/µm^2^, respectively, *p* < 0.0001, Fig. 2C, D). As with nanosilver, the transcriptomic data also indicated secondary defense changes in biofilm growth behavior, with upregulations of *bap* by ~8.6-fold and *bfmR* by ~2.8-fold in Ag^T^, relative to WT, when exposed to ionic silver (0.5 × MIC) (Fig. 2B). No statistically significant upregulations of these genes were detected in the WT strain upon ionic silver exposure, relative to the cell-only samples. Without silver, a ~1.6-fold *pgaAB*, ~8.2-fold *bap* and ~3.1-fold *bfmR* upregulations were detected in Ag^T^, relative to WT (Fig. 2B).

The observations revealed changes in biofilm growth behavior in response to long-term silver exposure. While previous studies have reported elevated biofilm growth in antibiotic-resistant bacteria, to the best of our knowledge, this is the first study to link stably enhanced surface-attached colonization and EPS formation traits in a bacterium following evolutionary adaptation to antimicrobial silver^19,39^. With comparable transcriptomic and phenotypic trends, only slight differences in biofilm growth characteristics between the silver-adapted strains were observed. A statistically significant (*p* < 0.001) increase in EPS formation was observed in the silver-exposed (0.5 × MIC) Ag^T^ biofilms compared to NAg^R^ (Fig. 2C, D). Next, we describe the evolved cellular defense responses to the well-established oxidative stress toxicity associated with silver.

### Oxidative stress defense and opportunistic silver efflux

**Fig. 3:**
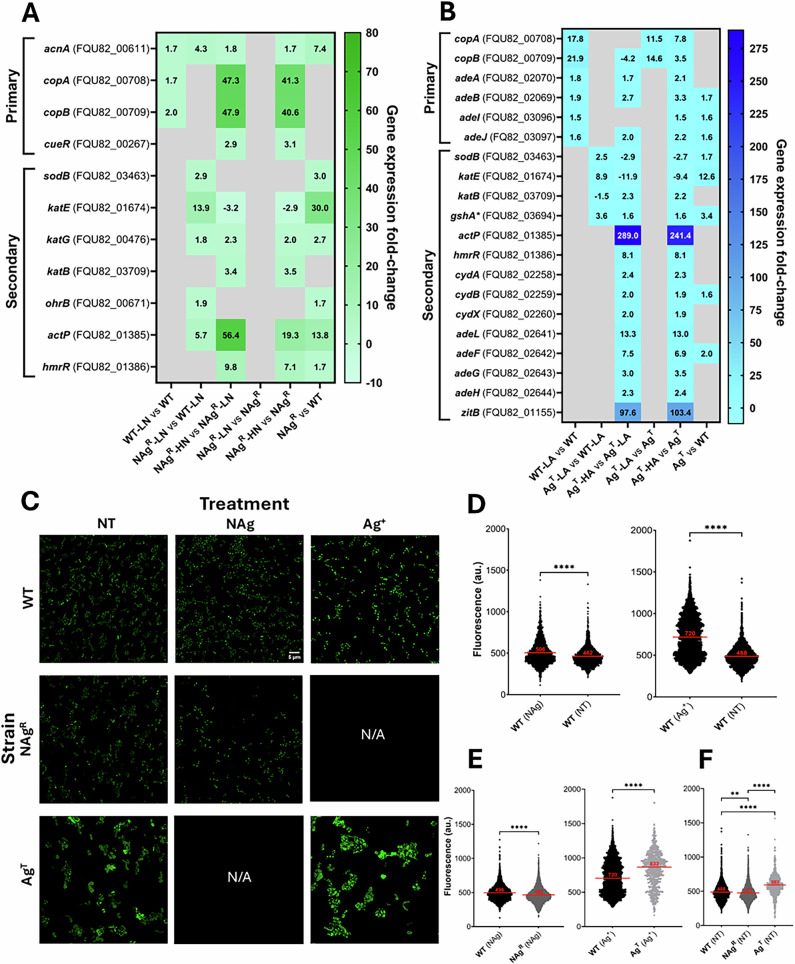
Oxidative stress management and efflux mechanisms in silver-adapted *A. baumannii.* **A**, **B** Comparative analysis of chromosomal mRNA levels of genes involved in ROS scavenging and oxidative stress response (*sodB, katE, katG, katB, ohrR, acnA*), as well as metal efflux (*copA, copB, cueR, actP, hmrR*) in WT, NAg^R^ and Ag^T^ after exposure to low silver concentrations (LN = ‘low’ nanosilver; 0.5 µg Ag/mL [0.5 × MIC], LA = ‘low’ ionic silver; 1 µg Ag/mL [0.5 × MIC] and high silver concentrations (HN = ‘high’ nanosilver; 3 µg Ag/mL [3 × MIC], HA = ‘high’ ionic silver; 3 µg Ag/mL [3 × MIC]). Note the exclusive upregulations of the respiratory chain components (*cydA*, *cydB*, *cydX*) and efflux systems (*adeA*, *adeB*, *adeI*, *adeJ*, *adeL*, *adeF*, *adeG*, *adeH*) in Ag^T^, not observed in NAg^R^. The (*) denotes detection of single nucleotide mutation in the oxidative stress control gene *gshA*. The differentially expressed genes (log_2_-fold change threshold ≥0.58 or ≥1.5-fold change; *p*-adjusted value ≤0.01; grey cells = statistically insignificant change [*p*-adj >0.01]) were captured at 30 min of silver exposures in the exponential growth phase. Also shown are the physiological gene expression changes when in the absence of silver. **C** Fluorescence microscopy images of cellular ROS generation (green) in the WT and the silver-adapted strains when exposed to silver (0.5 × MIC of NAg and Ag^+^; 30 min–1 h). Also shown are basal ROS levels generated in the respective cell-only (untreated) samples, scale bar = 5 µm. Quantitative analysis shows fluorescence intensity indicative of ROS levels in (**D**) silver-exposed WT (1 h treatment) to showcase silver-induced ROS generation, and in **E** NAg^R^ and Ag^T^ (30 min treatment) to indicate ROS management. Also shown in **F** is the physiological presence of cellular ROS in WT, NAg^R^ and Ag^T^, without silver. Each data point represents single cell measurements, with the horizontal bar showing the mean data from 1000 to 4000 cells analyzed, per strain, per treatment. Statistical analysis (unpaired *t*-test with Welch’s correction) shows statistically significant differences in cellular ROS levels between strains and treatments (****p < 0.0001). The mRNA and phenotypic work were performed with a minimum of three biological replicates.

As herein observed with the WT strain (0.5 × MIC, *p* < 0.0001, Fig. 3C, D), nanosilver is known to generate cellular oxygen radicals upon exposure to bacteria, which, among others, include the highly reactive one-electron reductant/oxidant superoxide (O_2_^•-^) and hydroxyl radicals (•OH) that target biomolecules^24,29^. NAg^R^ upregulated the superoxide dismutase *sodB* by ~2.9-fold and catalases *katE*, *katG* by ~14 and ~1.8-fold, respectively, relative to WT when exposed to the low nanoparticle concentration (0.5 × MIC) (*p*-adj ≤0.01, Fig. 3A). High nanoparticle concentration exposures (3 × MIC, 3 µg Ag/mL) caused further upregulation of *katG* by ~2.3-fold in NAg^R^, relative to the low concentration exposures. The resistant strain also increased the expression of other catalase *katB*, by ~3.4-fold, relative to the low concentration exposures. The enzyme superoxide dismutase SodB catalyzes the dismutation of superoxide radical to molecular O_2_ and H_2_O_2._ The enzymes KatE (a hydroperoxidase, HPII), KatG (HPI), and KatB, then catalyze the reduction of H_2_O_2_ to O_2_ and H_2_O^86^. This reduction step would prevent cellular H_2_O_2_ conversion into other oxygen radicals, for instance, into highly reactive ^•^OH, through the biologically common Fe(II)-mediated Fenton-type reaction^24^. Studies have reported upregulations of *katE* and *katG* in *A. baumannii* in response to H_2_O_2_ oxidative stress, with the bacterium thought to express the latter catalase with heightened oxidative stress levels, as seen with the high nanoparticle concentration exposures herein^86–88^. It is also worth noting that *katE* was downregulated at higher NAg concentrations, by ~3.2-fold, relative to the low concentration exposures (Fig. 3A). These upregulations of the ROS scavengers align with the lower detected levels of cellular ROS in NAg^R^, relative to WT, upon exposure to the nanoparticle (0.5 × MIC, *p* < 0.0001, Fig. 3C, E). The resistant strain also increased the expression of *ohrB* by ~1.9-fold, which encodes the organic hydroperoxide resistance protein OhrB, which also helps control cellular H_2_O_2_ levels, but only at the low nanoparticle concentration exposures (0.5 × MIC), relative to WT (Fig. 3A)^89,90^. Upregulations of these ROS scavengers were seen in NAg^R^, while absent in WT, and is hence defined as secondary defense mechanisms. The WT only increased the expression of *acnA* by ~1.7-fold under nanoparticle exposure (0.5 × MIC), relative to the cell-only samples (Fig. 3A). The enzyme aconitate hydratase AcnA has been indicated to maintain the citric acid cycle during (or recovery from) cellular oxidative stress in *A. baumannii*^65,85,91,92^. The resistant strain also upregulated *acnA*, by ~4.3-fold with the low nanoparticle concentration exposures (0.5 × MIC), relative to WT, and further, by ~1.8-fold with the high nanoparticle concentration exposures (3 × MIC), relative to the low concentration exposures (Fig. 3). Notably, almost all ROS scavengers, inclduing *sodB*, *katE*, *katG*, *ohrB* and *acnA* (excluding *katB*), were innately upregulated in NAg^R^, without the presence of silver, by ~1.7–30-fold, relative to the WT, which indicates they are of stable defense traits (Fig. 3A).

Next, we observed increased expressions of copper efflux genes in both WT and NAg^R^, believed to function as opportunistic silver defense mechanisms. Upregulation of *copA* and *copB* by ~1.7–2.0-fold was detected in WT, when exposed to nanosilver (0.5 × MIC), relative to the cell-only samples (Fig. 3A), and by ~47–48-fold in NAg^R^ following high nanoparticle concentration exposure (3 × MIC), relative to those at low concentrations. CopA and CopB are inner membrane-bound P-type ATPase copper (Cu^+^) efflux pumps, which reportedly confer cross-resistance to silver ions in bacteria^93,94^. Earlier studies have reported upregulations of *copA* in response to silver exposures^70,71,95^. Upregulation of these efflux pumps has also been linked to less cellular ROS generation with metal exposures, as observed herein^70,71,94–96^. In *A. baumannii*, *copA* (and potentially *copB*) expressions are regulated by the HTH-type transcriptional regulator CueR^94,96,97^. Indeed, in conjunction with the increased expression of *copA* (and *copB*) in the resistant strain, a ~2.9-fold upregulation of *cueR* was only seen in NAg^R^ in response to high nanoparticle concentration exposures (3 × MIC), relative to those at lower concentrations (0.5 × MIC) (Fig. 3A). Following high nanoparticle dose exposure, the resistant strain also increased the expression of another copper (trans-membrane) P-type ATPase efflux pump *actP*^98,99^, by ~56-fold, as well as its transcriptional regulator *hmrR*^94^, by ~9.8-fold, under relative to the low concentration. There were no statistically significant changes in the expression of *copA* or *copB* in Ag^T^ compared to WT in the absence of silver (Fig. 3A). This supports the idea that the upregulation of these efflux genes is induced by heavy metals, with silver being the specific inducer in this case^93,94,100^. As described later, physiological upregulations (without the presence of silver) were observed for efflux mechanisms, specifically for non-heavy metal-specific multidrug efflux pumps.

As in the case of nanosilver resistance, exposure to cationic silver also led to upregulations of the ROS scavenger superoxide dismutase and catalases, as well as the efflux pumps, in the tolerant strain (Ag^T^). Ag^T^ showed increased expressions of *sodB* and *katE* by ~2.5-fold and ~8.9-fold, respectively, relative to WT, when exposed to the low ionic silver concentration (0.5 × MIC, 1 µg Ag/mL) (Fig. 3B). At the higher silver concentrations (3 µg Ag/mL), Ag^T^ also upregulated *katB* by ~2.3-fold, relative to the low concentration exposures (0.5 × MIC). Like NAg^R^, Ag^T^ also downregulated *katE* at higher concentrations by ~12-fold, relative to the low concentration exposures. The tolerant strain increased expression of *copA* and *copB*, following low ionic silver concentration exposures (0.5 × MIC), by ~12- and 15-fold, respectively, and by ~7.8 and 3.5-fold, with the high silver concentration (3 µg Ag/mL), relative to the cell-only samples (Fig. 3B). Ag^T^ also upregulated *actP* at substantial levels, by ~290-fold, as well as *hmrR*, the transcriptional regulator, by ~8.1-fold, with the high silver concentration exposures (3 µg Ag/mL), relative to the low concentration (0.5 × MIC), as also observed with NAg^R^. Notably, *copA* and *copB* were also upregulated in WT upon exposure to ionic silver (0.5 × MIC), by ~18- and 22-fold, respectively, relative to the cell-only samples. The seemingly higher extent of *copA* and *copB* expression in WT than those in the ionic silver-adapted strain aligns with the non-observed upregulation of the transcriptional regulator *cueR* (in the latter strain). Interestingly, as later described, Ag^T^ upregulated various non-heavy metal-specific, multidrug efflux pump clusters, not seen in the resistant strain. In fact, despite similarities in ROS scavenger and efflux expression changes, phenotypically, Ag^T^ exhibited different cellular ROS generation profiles following (ionic) silver exposure, when compared to NAg^R^ following silver (nanoparticle) exposure. The tolerant strain also displayed unusual inherent cellular ROS profiles when in the absence of silver. As described in the next section, it is evident that the bacterium had also developed distinct defense mechanisms in response to ionic silver, particularly those related to efflux and oxidative stress control. This is thought to highlight, at least in part, the different adaptation characteristics of tolerance versus resistance that evolved in response to prolonged ionic versus nanoparticulate silver exposure. On a side note, when in the absence of silver, upregulations of *sodB* and *katE* were detected in Ag^T^, by ~1.7- and 13-fold, respectively, relative to WT, suggesting they function as stable defense mechanism (Fig. 3B). Again, as seen with NAg^R^, there were no statistically significant upregulations of *copA*/*copB* metal efflux genes in Ag^T^ in the absence of silver. In addition, the upregulation of *bmfR* in both NAg^R^ and Ag^T^ (Fig. 2A/B) may also relate to the observed transcriptomic changes in these oxidative stress management genes, particularly the catalase/antioxidant genes. As mentioned, BfmR(S) is a global transcriptional regulatory system, and studies highlight that *bfmR* is necessary for the expression of oxidative stress genes, such as *katE*, as well as multidrug efflux pumps, including the Ade cluster efflux systems (discussed further below), in *A. baumannii*^84,85,101^.

### Unique defense and physiological changes in ionic silver-tolerant strain

Recalling the earlier described overlapping (cell envelope and biofilm-associated) defense mechanisms related to nanosilver adaptation, this study also revealed that *A. baumannii* had evolved unique adaptation strategies to ionic silver. The tolerant strain exhibits distinct morphological and physiological traits. Firstly, Ag^T^ displayed irregular, elongated cell morphology in contrast to the well-defined coccobacilli shape of NAg^R^ (as well as WT) (Fig. 1C). We hypothesized there might be differences in membrane density between the silver-adapted strains. Using the lipophilic membrane dye FM4-64, a higher membrane fluorescence intensity (per µm of cell perimeter, *p* < 0.0001, Fig. 1C, D) was detected in Ag^T^ when compared to NAg^R^, potentially indicating the former exhibits a more denser membrane structure. Earlier studies have reported morphological transformation in bacteria, including in *A. baumannii*, as a stress response to both antibiotics and silver at sub-lethal levels^102–104^. Indeed, the morphologically changed Ag^T^ evolved from the WT following prolonged sub-lethal (ionic) silver. One mechanism possibly involved in these morphology changes may be OmpA. The outer membrane-embedded protein is anchored to the periplasmic peptidoglycan layer, and its upregulation, as seen in Ag^T^, has been indicated to affect this outer membrane-peptidoglycan interaction, altering cell shape^105,106^. It remains unclear as to why no detectable morphological changes were observed in NAg^R^, despite upregulation of *ompA* in this strain. Herein, with support from robust molecular evidence, Ag^T^ exhibited higher cellular ROS levels compared to both WT (*p* < 0.0001) and NAg^R^ (*p* < 0.0001) even in the absence of silver (Fig. 3C, F)—a trait commonly associated with antimicrobial-tolerant strains^107,108^^.^ Various studies have highlighted that tolerant bacterial populations can exhibit enhanced respiratory activity, hence the physiologically elevated cellular ROS levels in Ag^T^, as H_2_O_2_ is a natural by-product of respiration^107,108^. Note that this ROS build-up is independent of the silver-induced oxidative stress, also seen with exposures of WT strain to ionic silver (0.5 × MIC, 1 µg Ag/mL), relative to the cell-only samples (*p* < 0.0001, Fig. 3C, D). Consistent with the detected elevated ROS levels, this study observed increased expression of respiratory chain enzymes and efflux mechanisms in Ag^T^, which were unobserved in NAg^R^. Several of these distinct upregulations were also detected in Ag^T^ in the absence of silver, indicating that these are stable mechanisms.

Ag^T^ upregulated *cydABX* by ~2.0–2.4-fold, upon exposure to the high ionic silver concentration (3 µg Ag/mL), relative to the low concentration (0.5 × MIC, 1 µg Ag/mL) (*p*-adj ≤0.01, Fig. 3B). This gene cluster encodes the inner membrane-bound cytochrome *bd* oxidase complex CydABX, the terminal oxidase in the respiratory chain, which catalyzes O_2_ reduction into water and drives the proton motive force for ATP production^109^. Ionic silver (as well as ROS) has been indicated to target thiol groups in membrane-bound respiratory enzymes, inhibiting their activities and consequently disrupting the electron transport chain^24^. Upregulation of *cydABX* is thought to compensate for the Ag^+^-targeting of the respiratory chain, hence maintaining the enhanced respiration characteristics in the tolerant strain. No statistically significant change in *cydABX* expression was observed in Ag^T^, relative to WT, with the low concentration exposures (0.5 × MIC), suggesting it functions as a stress defense response to higher Ag^+^ levels. Furthermore, various multidrug and heavy metal efflux systems were upregulated in only Ag^T^ (and not NAg^R^), which may serve as additional mechanisms to protect the bacterium from Ag^+^ targeting. The tolerant strain upregulated multidrug efflux pumps, which included the two known RND (resistance-nodulation-division) efflux gene clusters in *A. baumannii*, *adeABC* and *adeIJK*. Ag^T^ showed increased expressions of *adeA* and *adeB* by ~1.7 and 2.7-fold, respectively, as well as *adeJ* by ~2.0-fold, upon exposure to the high ionic silver concentration (3 µg Ag/mL), relative to lower concentrations (0.5 × MIC, 1 µg Ag/mL) (Fig. 3B). In fact, upregulations of these genes were already observed in WT, at ~1.5–1.9-fold for *adeA*, *adeB*, *adeI* and *adeJ*, in response to ionic silver exposures (0.5 × MIC), relative to the cell-only samples. Studies show these efflux pumps have roles in antibiotic resistance in *A. baumannii*, with emerging evidence indicating they may also confer heavy metal resistance^110,111^. Also unique to Ag^T^, the strain upregulated the multidrug efflux pump cluster *adeFGH* by ~2.3–7.5-fold, as well as *adeL*, the transcriptional regulator, by ~13-fold, in response to high silver concentrations (3 µg Ag/mL), relative to the low concentration exposures (0.5 × MIC)^112^. On a more significant level, Ag^T^ upregulated *zitB*, which encodes the zinc(II) transporter ZitB, a member of the cation diffusion facilitator family, by ~98-fold, with the high silver concentration (3 µg Ag/mL), relative to the low concentration exposures (0.5 × MIC). Upregulations of *zitB* have been observed in bacteria in response to toxic zinc levels^113^. Interestingly, the overexpression of *zitB*, as observed here, has been shown to play a role in defense responses to other heavy metals, such as iron and cobalt. This suggests that the protein may have non-specific functions, potentially including silver transport in this case^114^. Our phenotypic cellular ROS observations in Ag^T^, when in the presence of silver, appear to align with the transcriptomic changes. Relative to WT, Ag^T^ was found with higher cellular ROS levels, when exposed to ionic silver (0.5 × MIC) (*p* < 0.0001, Fig. 3C, E). This contrasts with those earlier described with NAg^R^, whereby lower cellular ROS levels were detected in the resistant strain, relative to WT, under low nanosilver exposure (0.5 × MIC). The phenotypic findings support the hypothesized enhanced respiration characteristics in the tolerant strain, wherein ~1.6–2.0-fold stable upregulations of *cydB*, *adeB*, *adeI*, *adeJ* and *adeF* were detected in Ag^T^ (cell-only, Fig. 3B).

Ag^T^ upregulated a subset of ROS scavenger genes implicated in cellular ROS control but involved fewer genes than NAg^R^. Like the resistant strain, the tolerant strain upregulated the superoxide dismutase *sodB* and catalases *katE* and *katB* upon exposure to ionic silver (earlier described). The tolerant strain did not upregulate *katG* and *ohrB*, the latter of which also controls cellular H_2_O_2_ levels^89,90^, as well as *acnA*, which maintains the citric acid cycle (during or post oxidative stress)^65,85,91,92^. Notably, in our previous work, a single nucleotide mutation was observed in the glutamate-cysteine ligase gene *gshA* in Ag^T^, which is involved in the synthesis of the antioxidant glutathione (GSH)^115^. The substitutional (Gly39Arg) mutation was detected in 100% of the sequenced isolates^45^. While still unclear at this stage, the mutation is thought to invoke dysregulation of *gshA*, hence leading to increased expression by ~3.6-fold in Ag^T^, relative to WT, when exposed to the low ionic silver concentration (0.5 × MIC), and further, by ~1.6-fold in Ag^T^ in response to high silver concentrations (3 µg Ag/mL), relative to lower concentrations (Fig. 3B).

**Scheme 1:**
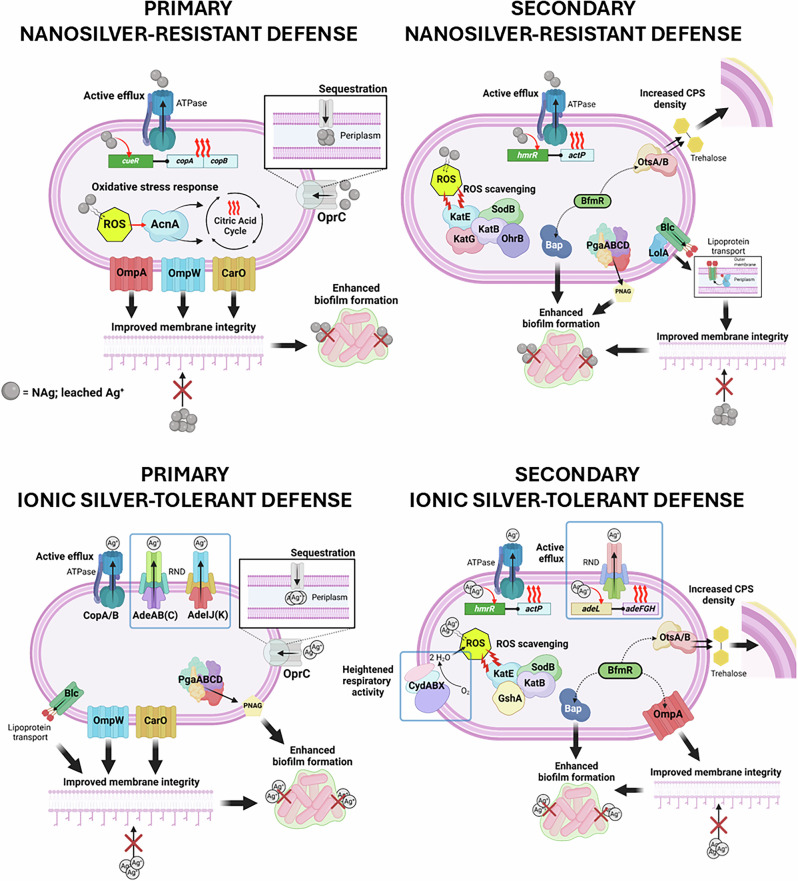
Working models of evolved silver defense mechanisms in the Gram-negative bacterium *A. baumannii*. The silver-adapted strains exhibit both primary and secondary mechanisms, with the wild-type only displaying primary mechanisms. Also shown (in blue frames) are the unique mechanisms that evolved in the tolerant strain specifically. Created in BioRender.

To summarize, we have established a working model for the modified defense mechanisms that evolved due to long-term nanosilver exposure (Scheme 1). The Gram-negative biofilm-forming bacterium developed stable primary defenses, those of which were already exhibited in the WT, as well as secondary defenses, which were only seen in the adapted strain. Cell surface defenses were activated following upregulations of (primary defense) outer membrane proteins, and membrane and capsule synthesis genes as secondary mechanisms. The phenotypically indicated increase in membrane integrity in the resistant strain is thought to also enhance the cell-to-surface adherence behavior of the bacterium, which could be associated with the observed increase in protective biofilm growth. We observed a higher incidence of surface-attached bacterial colonies, as well as greater abundance of the EPS matrix, with the resistant bacterium upregulating more cell-to-surface adherence genes and EPS synthesis genes, as part of its secondary defense. Finally, as anticipated, the bacterium had also evolved defenses to adapt to the known oxidative stress-mediated toxicity characteristics of nanosilver. ROS scavengers were upregulated as secondary mechanisms in the resistant bacterium, along with the predicted opportunistic silver efflux pumps, the latter of which were seen in the WT strain. The bacterium evolved different adaptation characteristics in response to long-term ionic silver exposure. The slower-to-kill Ag^T^ developed cell surface defenses that were generally comparable to those of NAg^R^, exhibiting similar upregulation patterns for outer membrane proteins (primary defense) and capsule synthesis genes (secondary defense). The tolerant strain also showed heightened biofilm growth behavior, with more surface-attached colony growth, which, like NAg^R^, is thought to associate with the phenotypically indicated increase in membrane protein expression. The tolerant strain, however, was found to form more EPS when compared to NAg^R^, although the reasons for this remain unclear. Indeed, Ag^T^ is physiologically different to NAg^R^. The tolerant strain exhibited higher cellular ROS profiles, which were linked to enhanced respirator activities – a recognized antimicrobial-tolerant trait. Not seen in NAg^R^, the tolerant strain upregulated the respiratory chain terminal oxidase cytochrome, as well as several multidrug and heavy metal efflux systems, thought to serve as defense mechanisms. More specifically, we predict they serve to replenish the respiratory chain component from known Ag^+^ targeting and to increase Ag^+^ expulsion, respectively. Ag^T^ also exhibited different oxidative stress control mechanisms, when compared to NAg^R^, overall upregulating less ROS scavengers or ROS control-associated genes. At this stage, the exact reasons for the distinct adaptation characteristics that evolved from long-term exposure to the nanoparticle, as opposed to the ionic form of silver, are unclear. Apart from their unique silver antimicrobial characteristics, the stable morphological changes seen with Ag^T^ could perhaps give us further clues about the different adaptation responses. Studies have suggested that bacteria change alter their shape to either increase their surface-to-volume ratio, hence enhancing the rate of nutrient uptake/antimicrobial efflux, or to decrease the ratio, therefore limiting antimicrobial influx^116,117^. As shown in Fig. 1C, the latter seems to apply for the tolerant strain which has a larger cell size or smaller ratio (relative to WT and NAg^R^), possibly to reduce the intake of silver ions.

Finally, the study uncovered a plasmid-associated stress response mechanism which could function against silver toxicity. Transcriptomic analysis of native plasmids in the bacterium (p1 of 7655 bp and p2 of 9540 bp) showed upregulations of the type II toxin/antitoxin (TA) gene *higBA* (present in both plasmids). Upon exposure to a high concentration of silver (3 µg Ag/mL), *higBA* expression increased by ~2.8–4.5-fold in NAg^R^ and 1.7–3.5-fold in Ag^T^, relative to both low-concentration (0.5 × MIC) exposures and untreated control samples (see Figs. S9 and S10)^107^ With respect to silver toxicity, studies have indicated that type II TA systems assist in modulating stress responses in bacteria, including following oxygen radical targeting^108,118^. While yet to be shown in *A. baumannii*, HigBA has been suggested to play a role in DNA repair. Studies with *Enterobacteriaceae* plasmids have highlighted the presence of LexA binding sites in the *higBA* promoter regions. LexA works together with RecA as an SOS response system, whereby binding of RecA to single strand (damaged) DNA will induce the self-release of LexA, which in this case, would lead to upregulation of *higBA*^119^. Both NAg^R^ and Ag^T^ upregulated *recA* by ~1.9 and 2.3-fold, respectively, at the high silver concentration, relative to the low concentration (0.5 × MIC) exposures (Table S4). HigBA has also been associated with enhanced biofilm growth in response to stressors, the latter of which manifested in the silver-adapted bacteria, as earlier described^108,119^.

To conclude, the molecular work presented here describes the adapted defense mechanisms of a Gram-negative, biofilm-forming bacterial pathogen that evolved in response to long-term nanosilver exposure, and further highlights how these mechanisms differ from those developed in response to ionic silver. The evolved ‘harder-to-kill’ nanosilver-resistant bacterium upregulated both ROS scavenger and heavy metal efflux mechanisms, as well as cell surface and biofilm defense systems. Specifically, these included various outer membrane proteins, along with membrane and capsule synthesis genes, for the latter. Enhanced biofilm formation was also observed, evidenced by a greater abundance of surface-attached colonies and the protective EPS matrix—also potentially linked to increased membrane density as supported by gene expression and phenotypic changes. These stable defense traits manifested as primary and secondary mechanisms, the latter of which were observed only in the silver-adapted strains, while absent in the parental wild-type bacterium. Despite similarities in cell surface and biofilm defense traits, the “slower-to-kill” ionic silver-tolerant strain upregulated respiratory chain enzymes and multidrug efflux systems. These exclusive mechanisms align with some scholarly evidence that antimicrobial-tolerant bacteria can exhibit enhanced respiratory characteristics, which in this case may potentially replenish the known cellular targets of Ag^+^, and increase cellular expulsion of the ions, respectively.

The findings highlight these unique antimicrobial characteristics and, therefore, distinct bacterial adaptation responses to the nanoparticle versus ionic forms of silver. While this study reveals extensive and complex transcriptional changes associated with silver exposure, a key limitation is the absence of RT-qPCR validation for gene expression. Incorporating RT-qPCR for future work, along with complementary molecular approaches such as gene knockout, overexpression mutant generation, or TraDIS, would strengthen the validity of these results. Nonetheless, this study provides a comprehensive, multi-layered effort to elucidate the complex nature of antimicrobial silver adaptation phenomena in bacteria at both molecular and phenotypic levels. Fully deciphering this evolution phenomenon is challenging, but these findings offer valuable insights into developing strategies to mitigate the risks associated with long-term silver exposure against bacteria. Notably, the observed increase in biofilm-forming capabilities could have important clinical implications, particularly in the context of silver-coated medical devices, which may reduce antimicrobial efficacy. Overall, the tiered defense mechanisms identified at the mRNA level may serve as potential molecular drug targets, helping to counteract resistance and uphold the effectiveness of these important nano-antimicrobials in the wake of the antimicrobial resistance era.

## Methods

### Silver nanoparticles and ionic silver agents

The silver nanoparticles (6.6 wt% Ag_2_O [*d*_TEM_ ≈ 2 nm] finely dispersed onto inert TiO_2_ [*d*_TEM_ ≈ 30 nm]) were synthesized via flame spray pyrolysis, as described by Gunawan et al.^40^. The nanoparticles were sterilized with gamma-irradiation (Cobalt-60) for 1 h at ~6 Gy/min dose at the Australian Nuclear Science Technology Organization (ANSTO). A fresh stock of NAg suspension was prepared in sterile cation-adjusted Mueller–Hinton broth (CAMHB; BD) and homogenized via ultra-sonication (20 s, 50% output; Vibra-cell, Sonics & Materials), prior to experimentation. Ionic silver was supplied as silver nitrate (AgNO_3_; Merck) suspended in sterile ultrapure Milli-Q water. All NAg and Ag^+^ exposure experiments were performed in dark conditions, to photo-catalytically inactivate the TiO_2_ support, and to prevent reduction of Ag^+^ to Ag^0^, respectively^25,40^.

### Bacterial strains and growth conditions

The wild-type (WT) *A. baumannii* ATCC 19606 strain used in this study was first isolated from a patient urine sample in 1948 by Schaub and Hauber^120^. The NAg-resistant (NAg^R^) and Ag^+^-tolerant (Ag^T^) strains were developed from ATCC 19606 through 30-day exposures to NAg and Ag^+^, respectively, in our previous study^45^. Frozen (–80 °C) glycerol stocks of the WT, NAg^R^, and Ag^T^ strains were streaked onto cation-adjusted Mueller–Hinton agar (CAMHA) plates and grown for 16-18 h at 37 °C. For all experiments, single isolated agar colonies were inoculated in CAMHB and grown overnight for 16-18 h at 37 °C, 250 rpm.

### Working concentrations of NAg and Ag^+^

To ensure minimal compensatory effects (as fitness cost trade-offs)^121^, we compared the early exponential phase growth rates of the WT, NAg^R^ and Ag^T^ strains with and without the NAg and/or Ag^+^ working concentrations. Briefly, overnight cultures of each strain were diluted in fresh CAMHB to OD_600_ 0.05, then cultured at 37 °C, 250 rpm for 2 h to achieve exponential (log) phase growth. The cultures were then exposed to the low (0.5 × MIC–0.5 µg Ag/mL for NAg, 1 µg Ag/mL for Ag^+^)^45^ and high (3 µg Ag/mL for NAg [3 × MIC] and Ag^+^ [1.5 × MIC])^45^ silver doses (Table S1), with hourly OD_600_ readings. Note that the different MIC-fold for the high NAg and Ag^+^ exposure concentrations were to ensure equivalent silver doses (3 µg Ag/mL). In addition, only the NAg^R^ and Ag^T^ strains were exposed to the high concentrations of silver, as such toxic silver levels would cause rapid killing in the WT, rendering transcriptomic analysis unfeasible in the latter strain^23,24,45^. The growth profiles are shown in Figs. S1 to S6, with comparable early exponential phase growth rates of the silver-exposed strains relative to their respective cell-only (i.e., silver-free or untreated) controls, hence establishing silver-induced effects. From hereafter, the mRNA and phenotypic studies were performed at the above-mentioned silver concentrations. The growth profile studies were performed with at least two biological replicates, each in technical triplicates.

### RNA extraction and sequencing

The low and high concentration silver-exposed and untreated cultures of the WT, NAg^R^ and Ag^T^ strains were harvested at 30 min of growth at 37 °C in the exponential phase (silver was added after 2 h growth, see above). The 30 min timeframe allowed direct profiling of the immediate silver-induced defense responses prior to the known extensive cell-killing activities at 1 h of exposure (at MIC level)^23,24^. See Figs. S1 to S6 for the OD_600_ readings of the silver-exposed and cell-only cultures at the time of RNA isolation. Total RNA extraction was performed using the RNeasy Mini Bacteria Kit (Qiagen) with RNAprotect Bacteria Reagent (Qiagen) following manufacturer’s instructions. Briefly, 0.5 mL of each of the silver-exposed (and cell-only) cultures was incubated with 1 mL RNAprotect (5 min, room temperature) and pelleted down (7500 rpm, 10 min). Cell pellets were lysed with lysozyme (Qiagen), with addition of Proteinase K (20 µL 10 mg/mL, Sigma-Aldrich), the latter to digest proteins and remove nucleases, for 15–20 min, room temperature (10 s vortex every 2 min), followed by DNase treatment for 15 min, room temperature. RNA extracts were then eluted from the spin column with RNase-free water (13,000 rpm, 1 min), followed by a dry spin. RNA quantity and purity was assessed using an Epoch microplate spectrophotometer (Agilent Technologies) and a Nanodrop spectrophotometer (Thermo-Fisher Scientific). RNA integrity was assessed using the TapeStation system with RNA Analysis ScreenTape (Agilent Technologies). All purified RNA samples contained RIN values of >8 (data not shown). Ribosomal RNA depletion, library preparation, and sequencing were performed at the Ramaciotti Centre for Genomics (UNSW, Australia) on a NovaSeq6000 platform (Illumina) generating up to 800 million single-end 100 bp reads.

### RNA-seq analysis

RNA-seq datasets (chromosomal and native plasmid transcripts) were analyzed using the limma-voom software^122^, in Galaxy Australia v3.58.1 (https://usegalaxy.org.au). The analysis pipelines, including quality control of reads via multi-dimensional principal component analysis (see Fig. S7), read coverage, counts and mapping, identification of differentially expressed genes (DEGs), and gene ontology clustering, are shown in Fig. S8, each with the software used. Briefly, the demultiplexed sequence reads underwent quality control analysis with FastQC. Trimmed and filtered reads (using Trimmomatic, allowing a minimum of 40-nt sequence) were aligned to the ATCC 19606 chromosome (GenBank accession number CP045110) and the two ATCC 19606 native plasmids, p1ATCC19606 and p2ATCC19606 (GenBank acc. no. CP045108 and CP045109) using the read mapper Bowtie2, allowing for one mismatch^107^. The frequency of mapped reads in each BAM file that aligned to the “gene” or “CDS” feature of the ATCC 19606 GFF3 file was calculated and used to generate tabular datasets for each sample using the count quantification software HTseq. The tabular datasets were used to identify DEGs between biological replicates and treatment conditions. The cut-off threshold to define a statistically significant DEG between pairwise comparisons was set at a log_2_-fold change of ≥0.58 (≥1.5-fold change) with an adjusted *p*-value of ≤0.01, as per the Benjamini and Hochberg false-discovery rate method^123^. Mapping of statistically significant DEGs to functional pathways with gene ontology was performed using ShinyGO (http://bioinformatics.sdstate.edu/go/) and the results are reported in Tables S2 and S3. Individual DEGs of interest associated with silver defense mechanisms of the WT, NAg^R^ and Ag^T^ strains were further examined. A summary outlining the silver exposure systems and details for each pairwise comparison (with total DEGs identified) is provided in Tables S4 and S5.

### Bacterial membrane microscopy

The WT, NAg^R^, and Ag^T^ strains were grown in the absence of silver. Upon reaching the exponential growth phase (2 h growth, see above), the cells were directly stained with FM 4-64 (10 μg/mL working concentration, Thermo-Fisher Scientific) for 10 min in the dark at room temperature. The stained cells were then pipetted onto 1.5% (w/v) agarose gel pads and imaged using DeltaVision (DV) Elite deconvolution fluorescence microscope (GE Healthcare), with mCherry filter (572/25 nm excitation, 632/60 nm emission) to visualize stained cell membranes. The images were analyzed with the Fiji (ImageJ) plugin MicrobeJ^124^.

### Biofilm microscopy

For biofilm growth, overnight cultures of WT, NAg^R^ and Ag^T^ strains were diluted to an OD_600_ of 0.05 in fresh CAMHB, inoculated into a FluoroDish (World Precision Instruments) and grown at 37 °C for 24 h in humidified conditions. Following removal of the supernatant, the surface-attached biofilms were washed twice with 1× phosphate-buffered saline (PBS). For silver exposures, the biofilms were treated with their respective silver agents (NAg and/or Ag^+^, 0.5 × MIC), for 24 h at 37 °C^45^. Untreated cultures for each strain were included. The cultures were washed twice with PBS, the biofilms were then stained with SYTO-9 (5 μM working concentration, Thermo-Fisher Scientific) and FilmTracer SYPRO Ruby (1× concentration per manufacture instructions, Thermo-Fisher Scientific) for 30 min at room temperature under dark condition. The stained biofilms were washed twice with PBS for imaging. Fluorescence imaging was performed using the DV Elite fluorescence microscope with FITC filter (475/28 nm excitation, 523/48 nm emission) for SYTO-9 (stained cells) and TRITC filter (632/22 nm excitation, 679/34 nm emission) for SYPRO Ruby (stained EPS/matrix proteins). All images were captured in sectional Z-stacks using the DV Elite SoftWoRx program and deconvolved using proprietary settings. The deconvolved biofilm images were analyzed using Imaris software v9.6.0 (Oxford Instruments) to determine colony biomass and EPS biomass (μm^3^/μm^2^) with automatic threshold (absolute intensity) detection applied. The 3D Z-stack images were compressed and presented as single plane images to provide visualization of the biofilm structures.

### Cellular ROS microscopy

The WT, NAg^R^ and Ag^T^ strains were first grown for 2 h (see above). The cells were pelleted, washed twice with PBS, re-suspended in sterile saline (8 g/L NaCl, 0.2 g/L KCl), and stained with ROS-reporter 2′,7′-dichlorodihydrofluorescein diacetate (H_2_DCFDA, 10 µM working concentration, Invitrogen) for 45 min at room temperature under dark conditions. The stained cultures were then pelleted, re-suspended in saline, and exposed to silver (NAg and/or Ag^+^, 0.5 × MIC) for 30 min and 60 min at 37 °C. Finally, the cultures were re-pelleted and re-suspended in saline. For imaging, the cells were pipetted into a Gene Frame (Thermo Fisher Scientific) containing a 2% (w/v) agarose gel pad. Cells treated with 50 mM hydrogen peroxide (H_2_O_2_) were used as a positive control. Fluorescence imaging was performed using the DV Elite microscope with FITC filter (475/28 nm excitation, 523/48 nm emission). The images were captured using the DV Elite SoftWoRx program and deconvolved. Five-to-six images were captured per biological triplicate, per strain and silver exposure system. For fluorescence quantitative analysis, background noise was subtracted with a rolling ball radius of 50 pixels and intensity values were calculated from individual cells using the Fiji (ImageJ) plugin MicrobeJ^124^.

## Supplementary information

Supplementary Information

## Data Availability

The RNA-seq datasets (chromosomal and native plasmid transcripts) that support the findings of this study are openly available in NIH National Center for Biotechnology Information at https://www.ncbi.nlm.nih.gov/bioproject/PRJNA557095/, BioProject accession number PRJNA557095.
