# Supplementary material for: Molecular insights into silver nanoparticle resistance in Acinetobacter baumannii and unique adaptations to ionic silver
Source: NPJ Antimicrob Resist. 2025 Nov 25;3:92. doi: 10.1038/s44259-025-00161-9 (PMC12647597; doi:10.1038/s44259-025-00161-9)
Supplement: Supplementary file 1 — Supplementary Information [file 44259_2025_161_MOESM1_ESM.docx]

Supporting Information

Molecular insights into silver nanoparticle resistance in *Acinetobacter baumannii* and unique adaptations to ionic silver

Oliver McNeilly, Daniel G. Mediati, Matthew J. Pittorino, Riti Mann, Bill Söderström,

Mehrad Hamidian, Cindy Gunawan

**Table S1. Determined working concentrations of NAg and Ag^+^ used to treat WT, NAg^R^, and Ag^T^ *A. baumannii* ATCC 19606 prior to RNA extraction**. DOI: 10.6084/m9.figshare.25955890

| **Treatment** | ***Acinetobacter baumannii* strain** | | |
| --- | --- | --- | --- |
|  | WT | NAg^R^ | Ag^T^ |
| **NAg treatment** | | | |
| Low NAg dose (sub-MIC, 0.5 x MIC) | 0.5 µg/mL | 0.5 µg/mL | - |
| High NAg dose (above MIC, 3 x MIC) | - | 3 µg/mL | - |
| **Ag^+^ treatment** | | | |
| Low Ag^+^ dose (sub-MIC, 0.5 x MIC) | 1 µg/mL | - | 1 µg/mL |
| High Ag^+^ dose (above-MIC, 1.5 x MIC) | - | - | 3 µg/mL |

**Table S2. Gene ontology enrichment analysis of statistically significant differentially expressed genes (DEGs) mapped to functional biological pathways identified in the RNA-seq comparisons of WT and NAg^R^ *A. baumannii* ATCC 19606.** DOI: 10.6084/m9.figshare.25956058

| **Enrichment FDR**^1^ | **No. of DEGs**^2^ | **No. of Pathway Genes**^3^ | **Fold Enrichment** | **Functional Pathway** |
| --- | --- | --- | --- | --- |
| **WTLA vs WTNT** | | | | |
| ***Upregulated*** | | | | |
| 0.003856942 | 1 | 4 | 713 | Aconitase/3-isopropylmalate dehydratase large subunit, alpha/beta/alpha domain |
| 0.003856942 | 1 | 4 | 713 | Aconitase/3-isopropylmalate dehydratase large subunit, alpha/beta/alpha, subdomain |
| 0.003856942 | 1 | 4 | 713 | Aconitase, iron-sulfur domain |
| 0.003856942 | 1 | 4 | 713 | Aconitase family (aconitate hydratase) |
| 0.004407934 | 1 | 5 | 356.5 | Mixed, incl. PrpF protein, and citrate synthase-like, small alpha subdomain |
| 0.004407934 | 1 | 4 | 356.5 | Aconitase/3-isopropylmalate dehydratase, swivel |
| 0.004407934 | 1 | 3 | 356.5 | Aconitase C-terminal domain |
| 0.034712482 | 1 | 49 | 39.61111111 | Mixed, incl. pyruvate, and tricarboxylic acid cycle |
| 0.04628331 | 1 | 72 | 26.40740741 | Mixed, incl. pyruvate, and NAD(p)-binding domain superfamily |
| ***Downregulated*** | | | | |
| N/A^4^ | | | | |
|  |  |  |  |  |
| **NRLN vs WTLN** | | | | |
| ***Upregulated*** | | | | |
| N/A | | | | |
| ***Downregulated*** | | | | |
| 0.007863504 | 4 | 4 | 6.79047619 | CF(1) |
| 0.000173287 | 6 | 8 | 6.79047619 | ATP synthesis, and ATP synthase A chain |
| 0.000173287 | 6 | 6 | 6.79047619 | ATP synthesis |
| 0.000173287 | 6 | 6 | 6.79047619 | Hydrogen ion transport |
| 0.04496184 | 3 | 5 | 6.79047619 | Mixed, incl. ribosomal protein S8, and ribosomal protein L1 |
| 0.006199586 | 5 | 6 | 5.658730159 | Mixed, incl. stress response, and Hsp90 protein |
| 0.029675761 | 4 | 5 | 5.432380952 | Translation protein SH3-like domain superfamily |
| 0.029675761 | 4 | 6 | 5.432380952 | Fatty acid biosynthesis |
| 1.61E-13 | 23 | 32 | 5.206031746 | rRNA-binding |
| 3.80E-21 | 37 | 60 | 5.024952381 | Ribonucleoprotein |
| 2.63E-21 | 38 | 61 | 4.962271062 | Ribosomal protein |
| 1.27E-20 | 38 | 67 | 4.778483245 | Ribosomal protein, and elongation factor Tu domain 2 |
| 1.04E-18 | 40 | 85 | 4.115440115 | Ribosomal protein, and elongation factor |
| 0.018354727 | 6 | 11 | 4.074285714 | Chaperone, and Hsp90 protein |
| 8.15E-20 | 44 | 102 | 3.983746032 | Ribosomal protein, and protein biosynthesis |
| 3.01E-20 | 54 | 161 | 3.333506494 | Mixed, incl. ribonucleoprotein, and protein biosynthesis |
| 6.29E-08 | 24 | 61 | 3.325947522 | RNA-binding |
|  |  |  |  |  |
| **NRHN vs NRNT** | | | | |
| ***Upregulated*** | | | | |
| 0.007760048 | 7 | 13 | 4.401234568 | Mixed, incl. histidine metabolism, and HutD |
| 0.038706688 | 5 | 6 | 4.401234568 | Histidine metabolism, and HutD |
| 0.000493409 | 11 | 29 | 4.034465021 | Dioxygenase |
| 0.007760048 | 9 | 108 | 3.601010101 | Mostly uncharacterized, incl. dioxygenase, and sugar (and other) transporter |
| 0.007760048 | 9 | 89 | 3.601010101 | Mixed, incl. dioxygenase, and sugar transporter, conserved site |
| 0.017992297 | 8 | 63 | 3.520987654 | Mixed, incl. dioxygenase, and coenzyme A transferase |
| 0.031939557 | 11 | 120 | 2.689643347 | Mixed, incl. LysR substrate binding domain, and LysE type translocator |
| 0.008084264 | 16 | 110 | 2.514991182 | Transcription regulation |
| 0.019823374 | 14 | 101 | 2.464691358 | Winged helix DNA-binding domain superfamily |
| 0.013984614 | 17 | 115 | 2.338155864 | Transcription |
| 0.017992297 | 17 | 125 | 2.267302656 | Winged helix-like DNA-binding domain superfamily |
| 0.007760048 | 21 | 174 | 2.254290876 | DNA-binding |
| ***Downregulated*** | | | | |
| 0.001954735 | 17 | 46 | 2.774954212 | Mixed, incl. ATP synthesis, and proton-conducting membrane transporter |
| 0.008435284 | 15 | 39 | 2.671078921 | Mixed, incl. ATP synthesis, and quinone |
| 0.042330184 | 12 | 36 | 2.611721612 | Protein biosynthesis |
| 0.014657461 | 19 | 74 | 2.255577756 | Ligase |
|  |  |  |  |  |
| **NRHN vs NRLN** | | | | |
| ***Upregulated*** | | | | |
| 0.008443366 | 7 | 13 | 4.570512821 | Mixed, incl. histidine metabolism, and HutD |
| 0.031438958 | 5 | 6 | 4.570512821 | Histidine metabolism, and HutD |
| 0.006785941 | 10 | 29 | 3.808760684 | Dioxygenase |
| 0.031438958 | 8 | 108 | 3.324009324 | Mostly uncharacterized, incl. dioxygenase, and sugar (and other) transporter |
| 0.031438958 | 8 | 89 | 3.324009324 | Mixed, incl. dioxygenase, and sugar transporter, conserved site |
| 0.029993603 | 11 | 53 | 2.793091168 | Mostly uncharacterized, incl. histidine metabolism, and nickel insertion |
| 0.029993603 | 11 | 120 | 2.793091168 | Mixed, incl. LysR substrate binding domain, and LysE type translocator |
| 0.027762545 | 14 | 101 | 2.559487179 | Winged helix DNA-binding domain superfamily |
| 0.027762545 | 15 | 110 | 2.448489011 | Transcription regulation |
| 0.029993603 | 16 | 115 | 2.28525641 | Transcription |
| 0.020356756 | 20 | 174 | 2.229518449 | DNA-binding |
| 0.031438958 | 16 | 125 | 2.216006216 | Winged helix-like DNA-binding domain superfamily |
| ***Downregulated*** | | | | |
| 0.021195614 | 16 | 46 | 2.41285956 | Mixed, incl. ATP synthesis, and proton-conducting membrane transporter |
| 0.021195614 | 20 | 74 | 2.193508691 | Ligase |
|  |  |  |  |  |
| **NRNT vs WTNT** | | | | |
| ***Upregulated*** | | | | |
| 0.03855924 | 11 | 181 | 3.267916667 | Signal |
| 0.000179797 | 33 | 235 | 2.262403846 | Oxidoreductase |
| ***Downregulated*** | | | | |
| 0.002725011 | 5 | 6 | 5.941666667 | Fatty acid biosynthesis |
| 0.012851779 | 5 | 6 | 4.951388889 | Mixed, incl. stress response, and Hsp90 protein |
| 0.012851779 | 5 | 8 | 4.951388889 | Fatty acid metabolism |
| 5.18E-23 | 40 | 59 | 4.850340136 | Ribosomal protein |
| 9.19E-23 | 40 | 60 | 4.753333333 | Ribonucleoprotein |
| 5.18E-23 | 42 | 67 | 4.621296296 | Ribosomal protein, and Elongation factor Tu domain 2 |
| 4.47E-12 | 23 | 32 | 4.555277778 | rRNA-binding |
| 2.05E-21 | 48 | 102 | 3.802666667 | Ribosomal protein, and protein biosynthesis |
| 0.042815578 | 6 | 11 | 3.565 | Chaperone, and Hsp90 protein |
| 2.24E-22 | 60 | 161 | 3.240909091 | Mixed, incl. ribonucleoprotein, and protein biosynthesis |
| 2.21E-07 | 25 | 61 | 3.031462585 | RNA-binding |

^1^ FDR = false discovery rate (adjusted *P*-value of 0.05). ^2^ Number of DEGs identified in functional pathway. ^3^ Number of total genes within functional pathway. ^4^ N/A = not applicable (no DEGs identified in pathway).

**Table S3. Gene ontology enrichment analysis of statistically significant differentially expressed genes (DEGs) mapped to functional biological pathways identified in the RNA-seq comparisons of WT and Ag^T^ *A. baumannii* ATCC 19606.** DOI: 10.6084/m9.figshare.26010916

| **Enrichment FDR**^1^ | **No. of DEGs**^2^ | **No. of Pathway Genes**^3^ | **Fold Enrichment** | **Functional Pathway** |
| --- | --- | --- | --- | --- |
| **WTLA vs WTNT** | | | | |
| ***Upregulated*** | | | | |
| 0.035818682 | 1 | 3 | 101.8571429 | Glycosyl transferase family 21 |
| 0.035818682 | 1 | 3 | 101.8571429 | Glycosyl transferase family group 2 |
| 0.035818682 | 1 | 5 | 101.8571429 | Glycosyltransferase like family 2 |
| 0.010080967 | 2 | 7 | 67.9047619 | Mixed, incl. PGAD-like protein, and hypothetical glycosyl hydrolase family 13 |
| 0.010080967 | 2 | 18 | 50.92857143 | Mixed, incl. glycosyltransferase like family 2, and glycoside hydrolase/deacetylase |
| 0.040082851 | 1 | 5 | 50.92857143 | Mixed, incl. NAD kinase, and Sir2 family |
| 0.040082851 | 1 | 6 | 50.92857143 | Mixed, incl. proton-conducting membrane transporter, and Na^+^/H^+^ antiporter subunit |
| 0.040082851 | 1 | 4 | 50.92857143 | NodB homology domain |
| 0.040082851 | 1 | 6 | 50.92857143 | Glycoside hydrolase/deacetylase, beta/alpha-barrel |
| 0.040082851 | 1 | 4 | 50.92857143 | Polysaccharide deacetylase |
| 0.022352934 | 2 | 81 | 18.51948052 | Mixed, incl. response regulator receiver domain, and histidine kinase A (phosphoacceptor) |
| 0.013792393 | 3 | 46 | 12.73214286 | Mixed, incl. ATP synthesis, and proton-conducting membrane transporter |
| 0.040082851 | 2 | 39 | 9.25974026 | Mixed, incl. ATP synthesis, and quinone |
| 0.040182649 | 2 | 181 | 8.488095238 | Signal |
| 0.013792393 | 5 | 617 | 4.428571429 | Transmembrane helix |
| 0.013792393 | 5 | 625 | 4.390394089 | Transmembrane |
| 0.022352934 | 5 | 668 | 3.663926002 | Membrane |
| ***Downregulated*** | | | | |
| 0.044394351 | 1 | 8 | 118.8333333 | Mixed, incl. protein of unknown function, DUF485, and sodium:solute symporter family |
| 0.044394351 | 1 | 7 | 118.8333333 | Mixed, incl. glyoxalase/fosfomycin resistance/dioxygenase domain, and fumarylace |
| 0.044394351 | 1 | 5 | 118.8333333 | Mixed, incl. citrate transporter, and Cyclin M, transmembrane domain |
| 0.013147271 | 2 | 4 | 59.41666667 | Histidine metabolism |
| 0.044394351 | 1 | 29 | 59.41666667 | Mostly uncharacterized, incl. phosphoesterase, and protein of unknown function |
| 0.044394351 | 1 | 11 | 59.41666667 | Mostly uncharacterized, incl. ligase N family, and citrate transporter |
| 0.044394351 | 1 | 5 | 59.41666667 | Glyoxalase/fosfomycin resistance/dioxygenase domain |
| 0.044394351 | 1 | 4 | 59.41666667 | Amidohydrolase-related |
| 0.044394351 | 1 | 4 | 59.41666667 | Metal-dependent hydrolase, composite domain superfamily |
| 0.044394351 | 1 | 6 | 59.41666667 | Glyoxalase/bleomycin resistance protein/dihydroxybiphenyl dioxygenase |
| 0.044394351 | 1 | 6 | 59.41666667 | Vicinal oxygen chelate (VOC) domain |
| 0.044394351 | 1 | 5 | 59.41666667 | Glyoxalase/Bleomycin resistance protein/Dioxygenase superfamily |
| 0.044394351 | 1 | 5 | 59.41666667 | Amidohydrolase family |
| 0.013147271 | 2 | 6 | 47.53333333 | Histidine metabolism, and HutD |
| 0.01826809 | 2 | 13 | 33.95238095 | Mixed, incl. histidine metabolism, and HutD |
| 0.044394351 | 2 | 53 | 13.2037037 | Mostly uncharacterized, incl. histidine metabolism, and nickel insertion |
|  |  |  |  |  |
| **ATLA vs WTLA** | | | | |
| ***Upregulated*** | | | | |
| 0.01775664 | 4 | 14 | 7.059405941 | Mixed, incl. thiosulphate/sulfate-binding protein, and phosphoadenosine phosphosulfate |
| 0.01775664 | 4 | 34 | 7.059405941 | Mixed, incl. alcohol dehydrogenase groES-like domain, and iron-type alcohol dehydrogenase |
| 0.016173219 | 5 | 20 | 5.882838284 | Mixed, incl. ion transport, and cation transporter |
| 0.000538968 | 9 | 68 | 5.294554455 | Mostly uncharacterized, incl. NMT1-like family, and MetI-like superfamily |
| 0.009938189 | 6 | 42 | 5.294554455 | Mostly uncharacterized, incl. molybdenum cofactor biosynthesis, and molybdopterin |
| 0.001534206 | 8 | 37 | 5.134113411 | Short chain dehydrogenase |
| 0.001534206 | 8 | 34 | 5.134113411 | KR domain |
| 0.000423979 | 10 | 85 | 5.042432815 | Mostly uncharacterized, incl. MetI-like superfamily, and NMT1-like family |
| 0.034530239 | 5 | 36 | 5.042432815 | Mixed, incl. TonB-dependent receptor-like, beta-barrel, and ACP-like superfamily |
| 0.005602223 | 7 | 32 | 4.941584158 | Short-chain dehydrogenase/reductase SDR |
| 0.005602223 | 7 | 21 | 4.941584158 | Short-chain dehydrogenase/reductase, conserved site |
| 0.005602223 | 7 | 34 | 4.941584158 | Enoyl-(Acyl carrier protein) reductase |
| 0.000210346 | 14 | 181 | 4.117986799 | Signal |
| 0.021188637 | 11 | 85 | 2.876054272 | NAD(P)-binding domain superfamily |
| 0.01775664 | 27 | 235 | 1.832730388 | Oxidoreductase |
| ***Downregulated*** | | | | |
| 0.002219513 | 4 | 4 | 8.695121951 | Histidine metabolism |
| 0.016622938 | 3 | 5 | 8.695121951 | Mixed, incl. ribosomal protein S8, and ribosomal protein L1 |
| 0.008946135 | 4 | 6 | 6.956097561 | Histidine metabolism, and HutD |
| 2.22E-16 | 23 | 32 | 6.666260163 | rRNA-binding |
| 3.73E-26 | 37 | 60 | 6.434390244 | Ribonucleoprotein |
| 1.77E-26 | 38 | 61 | 6.35412758 | Ribosomal protein |
| 0.004039135 | 5 | 13 | 6.210801394 | Mixed, incl. histidine metabolism, and HutD |
| 7.38E-26 | 38 | 67 | 6.118789521 | Ribosomal protein, and elongation factor Tu domain 2 |
| 2.18E-23 | 43 | 118 | 4.732787897 | Ribosomal protein, and protein biosynthesis |
| 9.41E-24 | 46 | 131 | 4.444173442 | Mixed, incl. ribonucleoprotein, and protein biosynthesis |
| 1.12E-10 | 24 | 61 | 4.258835241 | RNA-binding |
|  |  |  |  |  |
| **ATHA vs ATNT** | | | | |
| ***Upregulated*** | | | | |
| 3.80E-08 | 27 | 32 | 2.556573705 | rRNA-binding |
| 3.90E-05 | 19 | 39 | 2.453277798 | Mixed, incl. ATP synthesis, and quinone |
| 2.03E-08 | 40 | 60 | 2.27250996 | Ribonucleoprotein |
| 2.55E-08 | 39 | 59 | 2.260915522 | Ribosomal protein |
| 1.45E-07 | 40 | 67 | 2.104175889 | Ribosomal protein, and elongation factor Tu domain 2 |
| 5.19E-06 | 35 | 61 | 2.02902675 | RNA-binding |
| 3.24E-08 | 54 | 118 | 1.941701548 | Ribosomal protein, and protein biosynthesis |
| 6.88E-07 | 45 | 85 | 1.936798261 | Ribosomal protein, and elongation factor |
| 3.24E-08 | 61 | 138 | 1.863213811 | Mixed, incl. ribonucleoprotein, and protein biosynthesis |
| ***Downregulated*** | | | | |
| 0.04735129 | 9 | 84 | 3.363207547 | Mixed, incl. TonB dependent receptor, and protein transport |
| 0.04735129 | 10 | 32 | 3.203054807 | Mixed, incl. pseudouridine synthase, catalytic domain superfamily, and s-adenosylmethionine |
|  |  |  |  |  |
| **ATHA vs ATLA** | | | | |
| ***Upregulated*** | | | | |
| 3.51E-08 | 27 | 32 | 2.619183673 | rRNA-binding |
| 0.000252326 | 18 | 39 | 2.381076067 | Mixed, incl. ATP synthesis, and quinone |
| 2.08E-08 | 39 | 59 | 2.316284881 | Ribosomal protein |
| 2.63E-08 | 39 | 60 | 2.269959184 | Ribonucleoprotein |
| 7.57E-08 | 40 | 67 | 2.155706727 | Ribosomal protein, and elongation factor Tu domain 2 |
| 1.28E-05 | 34 | 61 | 2.019325281 | RNA-binding |
| 2.92E-07 | 45 | 85 | 1.984230056 | Ribosomal protein, and elongation factor |
| 4.39E-08 | 53 | 118 | 1.952415397 | Ribosomal protein, and protein biosynthesis |
| 1.62E-07 | 57 | 131 | 1.843129252 | Mixed, incl. ribonucleoprotein, and protein biosynthesis |
| ***Downregulated*** | | | | |
| N/A^4^ | | | | |
|  |  |  |  |  |
| **ATNT vs WTNT** | | | | |
| ***Upregulated*** | | | | |
| 0.0102631 | 5 | 20 | 5.991596639 | Mixed, incl. ammonium/urea transporter, and GlnD PII-uridylyltransferase |
| 0.002466615 | 6 | 20 | 5.991596639 | Mixed, incl. ion transport, and cation transporter |
| 0.030271401 | 4 | 14 | 5.991596639 | Mixed, incl. thiosulphate/sulfate-binding protein, and phosphoadenosine phosphosulfate |
| 0.030271401 | 4 | 34 | 5.991596639 | Mixed, incl. alcohol dehydrogenase groES-like domain, and iron-type alcohol dehydrogenase |
| 0.002466615 | 7 | 42 | 5.242647059 | Mostly uncharacterized, incl. molybdenum cofactor biosynthesis, and molybdopterin |
| 0.0102631 | 6 | 36 | 5.135654262 | Mixed, incl. TonB-dependent receptor-like, beta-barrel, and ACP-like superfamily |
| 0.000136111 | 10 | 68 | 4.992997199 | Mostly uncharacterized, incl. NMT1-like family, and MetI-like superfamily |
| 0.000136111 | 11 | 85 | 4.707683073 | Mostly uncharacterized, incl. MetI-like superfamily, and NMT1-like family |
| 0.0102631 | 8 | 84 | 3.994397759 | Mixed, incl. TonB dependent receptor, and protein transport |
| 0.02602932 | 7 | 37 | 3.812834225 | Short chain dehydrogenase |
| 0.02602932 | 7 | 34 | 3.812834225 | KR domain |
| 0.000136111 | 15 | 181 | 3.744747899 | Signal |
| 0.02602932 | 12 | 85 | 2.662931839 | NAD(P)-binding domain superfamily |
| 0.014492569 | 31 | 235 | 1.78595669 | Oxidoreductase |
| ***Downregulated*** | | | | |
| 0.036616266 | 3 | 5 | 7.427083333 | Mixed, incl. ribosomal protein S8, and ribosomal protein L1 |
| 0.005481389 | 4 | 4 | 7.427083333 | Histidine metabolism |
| 0.022426063 | 4 | 6 | 5.941666667 | Histidine metabolism, and HutD |
| 5.88E-23 | 37 | 59 | 5.608205782 | Ribosomal protein |
| 6.89E-23 | 37 | 60 | 5.496041667 | Ribonucleoprotein |
| 0.011370031 | 5 | 13 | 5.305059524 | Mixed, incl. histidine metabolism, and HutD |
| 7.59E-12 | 21 | 32 | 5.198958333 | rRNA-binding |
| 3.18E-21 | 37 | 67 | 5.088927469 | Ribosomal protein, and elongation factor Tu domain 2 |
| 1.52E-17 | 40 | 102 | 3.961111111 | Ribosomal protein, and protein biosynthesis |
| 1.40E-18 | 45 | 131 | 3.713541667 | Mixed, incl. ribonucleoprotein, and protein biosynthesis |
| 2.35E-06 | 21 | 61 | 3.183035714 | RNA-binding |

^1^ FDR = false discovery rate (adjusted *P*-value of 0.05). ^2^ Number of DEGs identified in functional pathway. ^3^ Number of total genes within functional pathway. ^4^ N/A = not applicable (no DEGs identified in pathway).

**Table S4. Number of DEGs (upregulated and downregulated genes) identified in WT and NAg^R^ *A. baumannii* treated with a low dose (0.5 µg/mL) and/or high dose (3 µg/mL) of NAg.** DOI: 10.6084/m9.figshare.26011252

| **Pairwise comparison** | **Upregulated genes** | **Downregulated genes** | **Total** |
| --- | --- | --- | --- |
| WTLN ^1^ versus WTNT ^2^ | 29 | 5 | 34 |
| NRLN ^3^ versus WTLN | 559 | 562 | 1121 |
| NRLN versus NRNT ^4^ | 5 | 2 | 7 |
| NRHN ^5^ versus NRNT | 1004 | 793 | 1797 |
| NRHN versus NRLN | 1013 | 821 | 1834 |

^1^ WTLN = wild-type low nanosilver; ^2^ WTNT = wild-type no treatment; ^3^ NRLN = nanosilver-resistant low nanosilver; ^4^ NRNT = nanosilver-resistant no treatment; ^5^ NRHN = nanosilver-resistant high nanosilver

**Table S5. Number of DEGs (upregulated and downregulated genes) identified in WT and Ag^T^ *A. baumannii* treated with a low dose (0.5 µg/mL) and/or high dose (3 µg/mL) of NAg.** DOI: 10.6084/m9.figshare.26011270

| **Pairwise Comparison** | **Upregulated genes** | **Downregulated genes** | **Total** |
| --- | --- | --- | --- |
| WTLA ^1^ versus WTNT ^2^ | 71 | 53 | 124 |
| ATLA ^3^ versus WTLA | 660 | 542 | 1202 |
| ATLA versus ATNT ^4^ | 8 | 0 | 8 |
| ATHA ^5^ versus ATNT | 1110 | 1011 | 2121 |
| ATHA versus ATLA | 1098 | 1000 | 2098 |

^1^ WTLA = wild-type low Ag^+^; ^2^ WTNT = wild-type no treatment; ^3^ ATLA = Ag^+^-tolerant low Ag^+^; ^4^ ATNT = Ag^+^-tolerant no treatment; ^5^ ATHA = Ag^+^-tolerant high Ag^+^


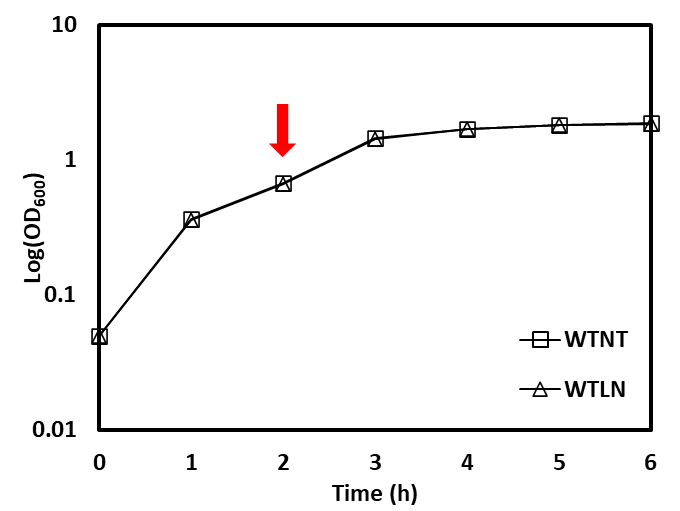


**Figure S1. Comparative growth rates of WT *A. baumannii* ATCC 19606 when treated with low NAg concentration (0.5 µg/mL; 0.5 x MIC) and untreated (no NAg) over 6 h.** WTNT = wild-type no treatment (square); WTLN = wild-type low NAg (triangle). The red arrow indicates the time point (2 h) at which NAg was added to (WTLN) culture. RNA was extracted 30 min after addition of NAg (2.5 h time point). Initial OD_600_ at time 0 began at 0.05. Each data point represents mean of at least two biological replicates. DOI: 10.6084/m9.figshare.26011282


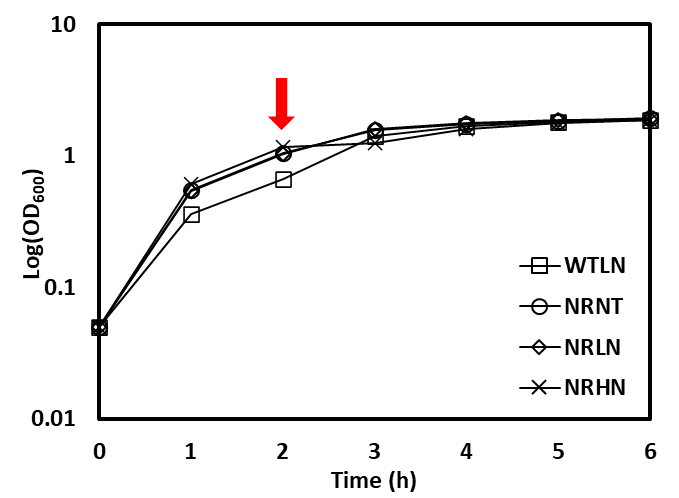


**Figure S2. Comparative growth rates of WT and NAg^R^ ATCC 19606 strains treated with low (0.5 µg/mL) and high (3 µg/mL) NAg doses over 6 h.** WTLN = wild-type no treatment (square); NRNT = NAg-resistant no treatment (circle); NRLN = NAg-resistant low NAg (diamond); NRHN = NAg-resistant high NAg (cross). The red arrow indicates the time point (2 h) at which NAg was added to respective cultures. RNA was extracted 30 min after addition of NAg (2.5 h time point). Initial OD_600_ at time 0 began at 0.05. Each data point represents mean of at least two biological replicates. DOI: 10.6084/m9.figshare.26011540


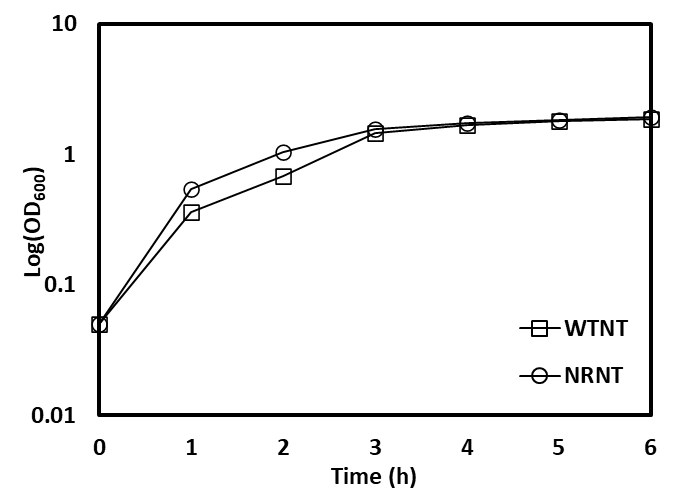


**Figure S3. Comparative growth rates of untreated (no NAg) WT and NAg^R^ ATCC 19606 strains over 6 h.** WTNT = wild-type no treatment (square); NRNT = NAg-resistant no treatment (circle). RNA was extracted at the 2.5 h time point. Initial OD_600_ at time 0 began at 0.05. Each data point represents mean of at least two biological replicates. DOI: 10.6084/m9.figshare.26011552


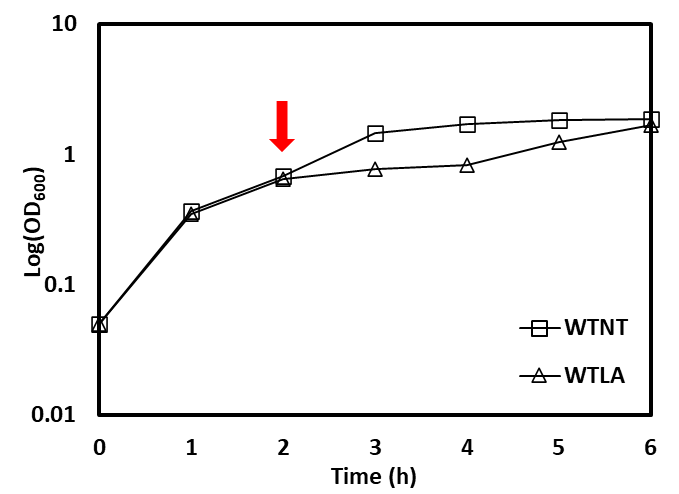

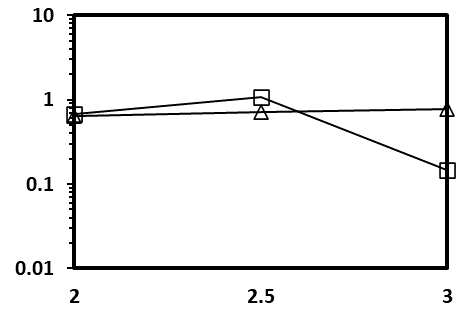


**Figure S4. Comparative growth rates of WT ATCC 19606 strain when treated with low Ag^+^ concentration (1 µg/mL) and untreated (no Ag^+^) WT ATCC 19606 over 6 h**. WTNT = wild-type no treatment (square); WTLA = wild-type low Ag^+^ (triangle). The red arrow indicates the time point (2 h) at which Ag^+^ was added to culture. RNA was extracted 30 min following addition of Ag^+^ (2.5 h time point). The inset plot displays an expanded time range from 2 – 3 h to show the closely comparable growth profiles of the untreated and treated bacterium. Initial OD_600_ at time 0 began at 0.05. Each data point represents mean of at least two biological replicates. DOI: 10.6084/m9.figshare.26011558


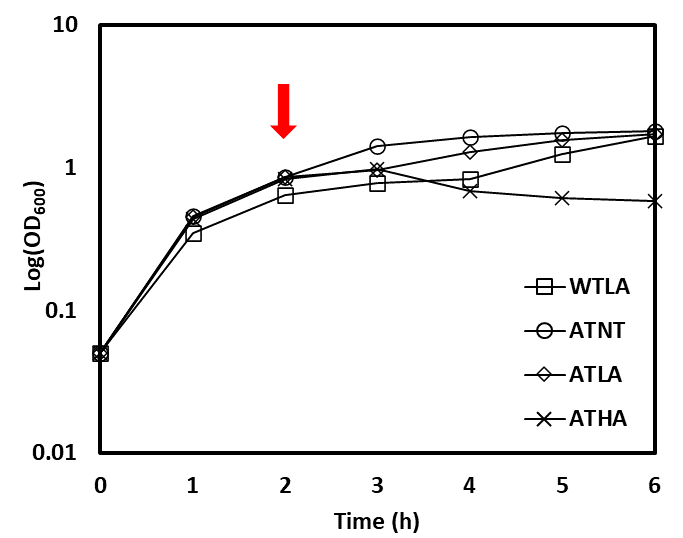

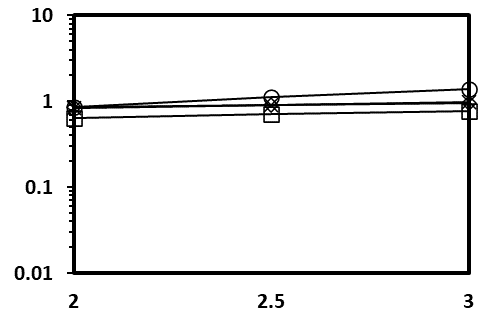


**Figure S5. Comparative growth rates of WT and Ag^T^ ATCC 19606 strains treated with low (1 µg/mL) and high Ag^+^ (3 µg/mL) doses over 6 h.** WTLA = wild-type low Ag^+^ (square); ATNT = Ag^+^-tolerant no treatment (circle); ATLA = Ag^+^-tolerant low Ag^+^ (diamond); ATHA = Ag^+^-tolerant high Ag^+^ (cross). The red arrow indicates the time point (2 h) at which Ag^+^ was added to the respective cultures. RNA was extracted 30 min after the addition of Ag^+^ (2.5 h time point). The inset plot displays an expanded time range from 2 – 3 h to show the closely comparable growth profiles of each culture. Initial OD_600_ at time 0 began at 0.05. Each data point represents mean of at least two biological replicates. DOI: 10.6084/m9.figshare.26011567


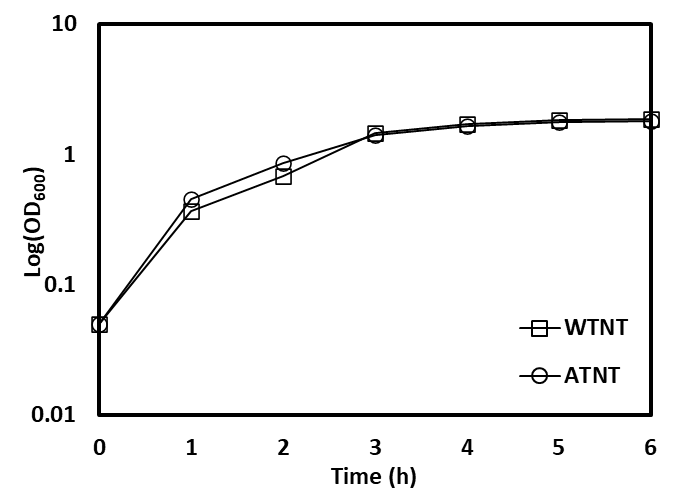


**Figure S6. Comparative growth rates of untreated (no Ag^+^) WT and Ag^T^ ATCC 19606 strains over 6 h.** WTNT = wild-type no treatment (square); ATNT = Ag^+^-tolerant no treatment (circle). RNA was extracted at the 2.5 h time point. Initial OD_600_ at time 0 began at 0.05. Each data point represents the mean of at least two biological replicates. DOI: 10.6084/m9.figshare.26011570

**Figure S7.** **Principal component analysis (PCA) .** The level of variation between biological triplicates of each ATCC 19606 strain and respective treatment type is displayed. Individual **
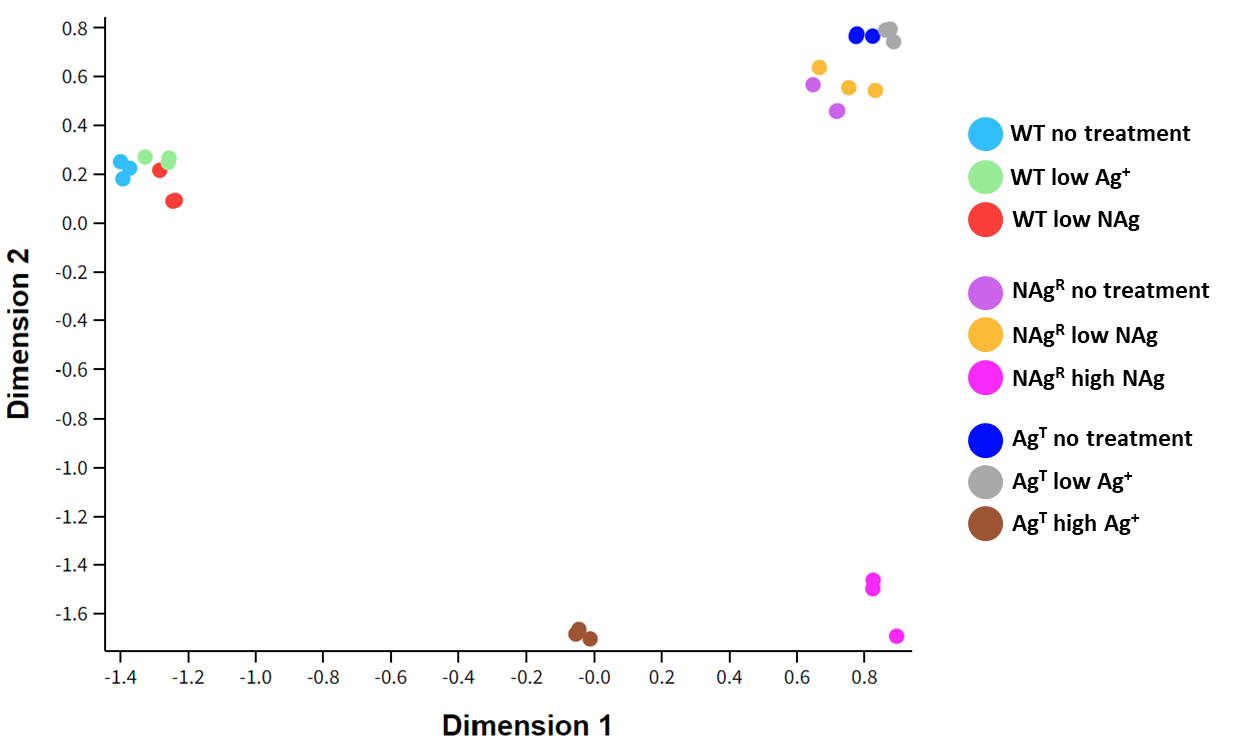
**coloured dots represent one of three biological replicates of population (and treatment) cluster. WT = wild-type strain, NAg^R^ = NAg-resistant strain, Ag^T^ = Ag^+^-tolerant strain. DOI: 10.6084/m9.figshare.26011570


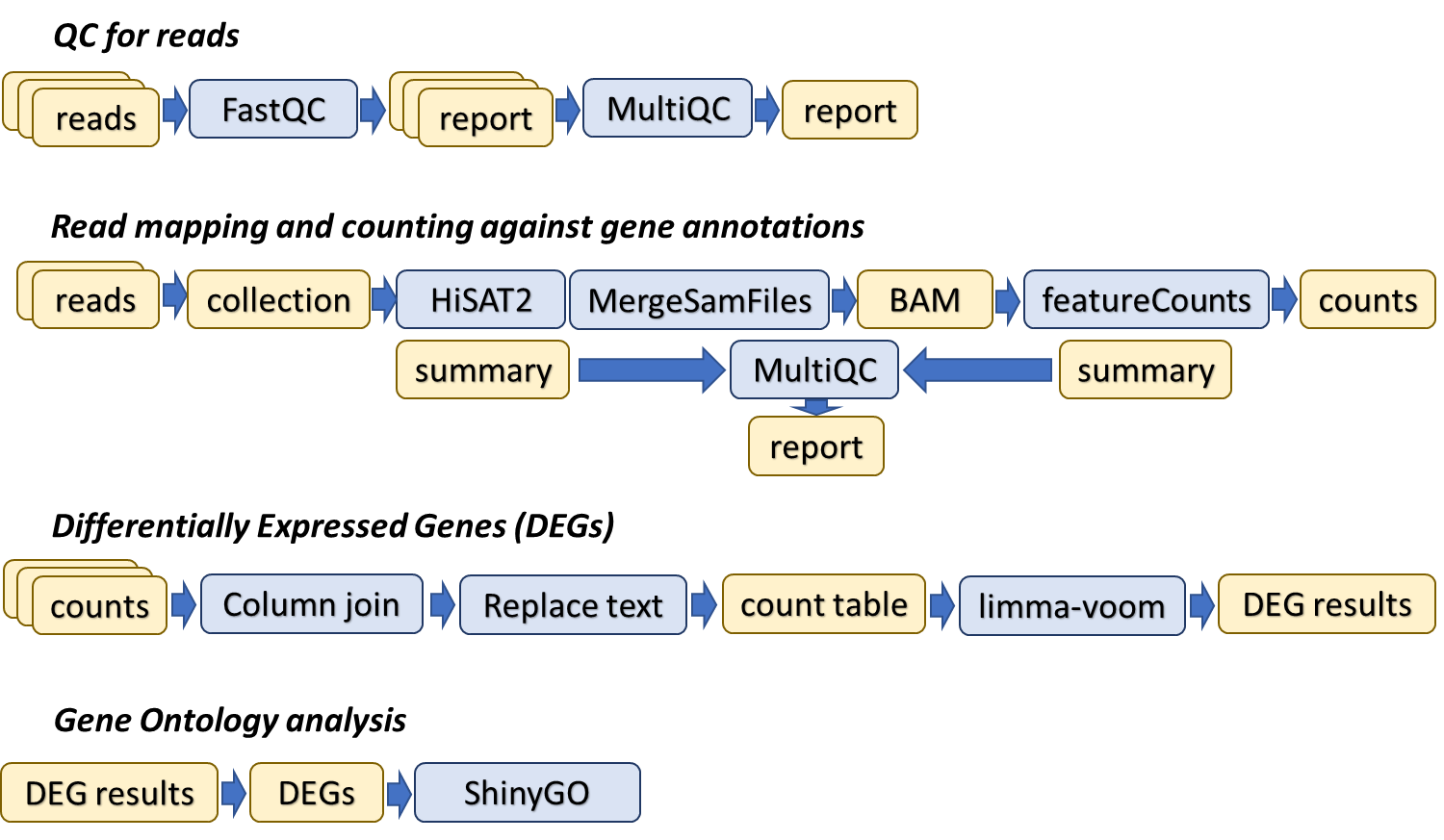
**Figure S8. Schematic workflow summary of RNA-seq** **study.** Analysis and data mapping was performed in Galaxy Australia. Blue arrows indicate order of events starting from top (QC for reads) to bottom (Gene Ontology analysis). DOI: 10.6084/m9.figshare.25955929


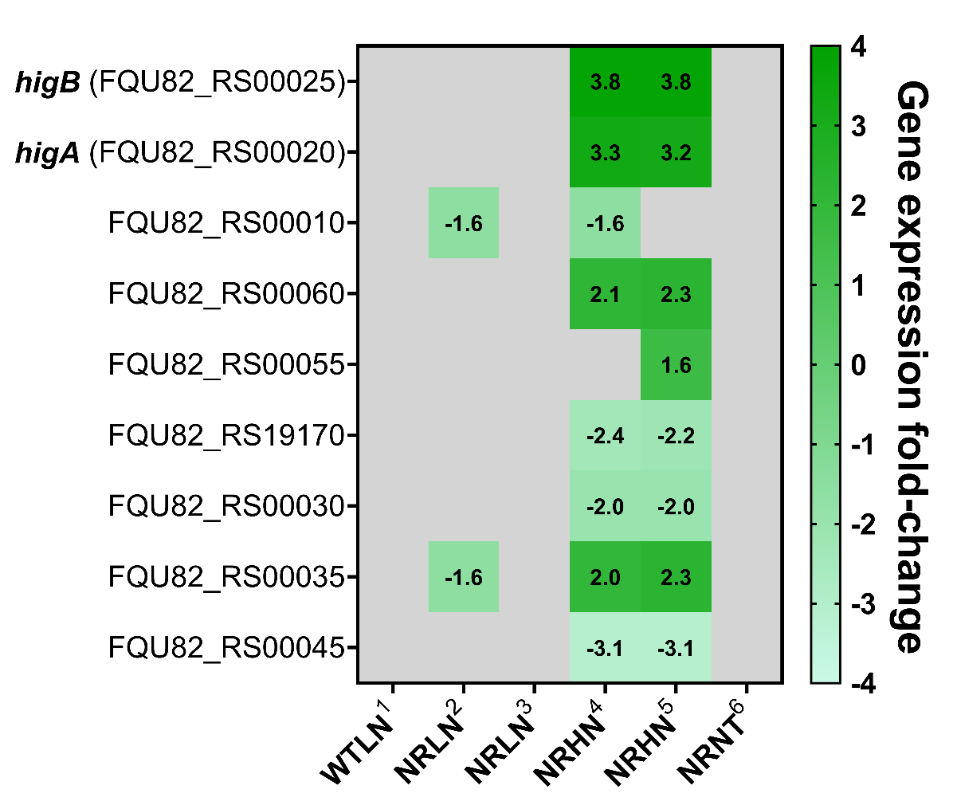


**A**


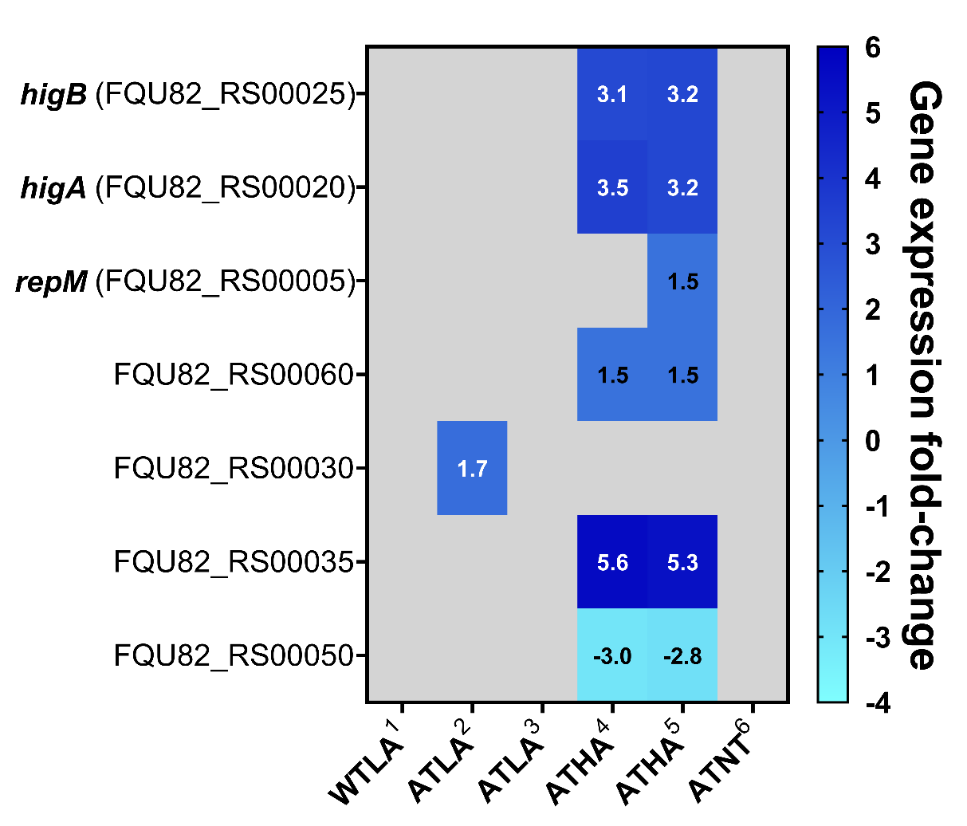


**B**

**Figure S9. Differentially expressed genes (DEGs) identified in plasmid p1 of WT, NAg^R^, and Ag^T^ *A. baumannii* ATCC 19606.** (**A**) Heat-map shows statistically significant (adj-*P* < 0.01) expression fold-change in plasmid-oriented genes in the WT and NAg^R^ strains. Pairwise comparison: ^1^ WTLN versus WTNT, ^2^ NRLN versus WTLN, ^3^ NRLN versus NRNT, ^4^ NRHN versus NRNT, ^5^ NRHN versus NRLN. (**B**) Statistically significant plasmid-oriented gene expression fold-changes in the WT and Ag^T^ strains. Pairwise comparisons: ^1^ WTLA versus WTNT, ^2^ ATLA versus WTLA, ^3^ ATLA versus ATNT, ^4^ ATHA versus ATNT, ^5^ ATHA versus ATLA, ^6^ ATNT vs WTNT. Genes with only locus tags provided are currently uncharacterised and have no known putative function. Fold-change values and scale (*right*) are included. Grey cells indicate statistically insignificant (adj-*P* > 0.01) fold-change for respective gene/mRNA transcript. NRNT and ATNT are the cell-only control cultures of the NAg^R^ and Ag^T^ strains, respectively. DOI: 10.6084/m9.figshare.26011591


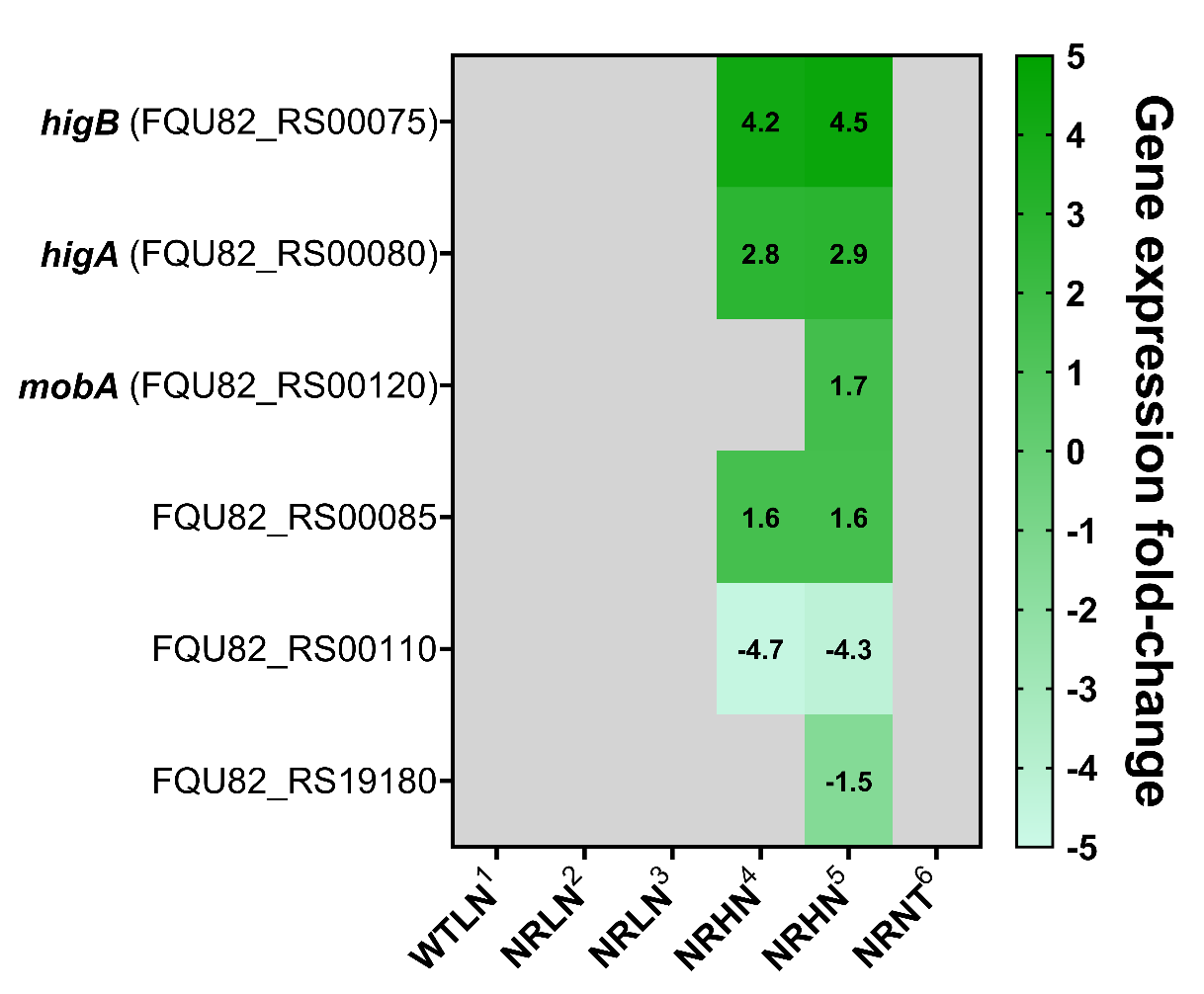


**A**


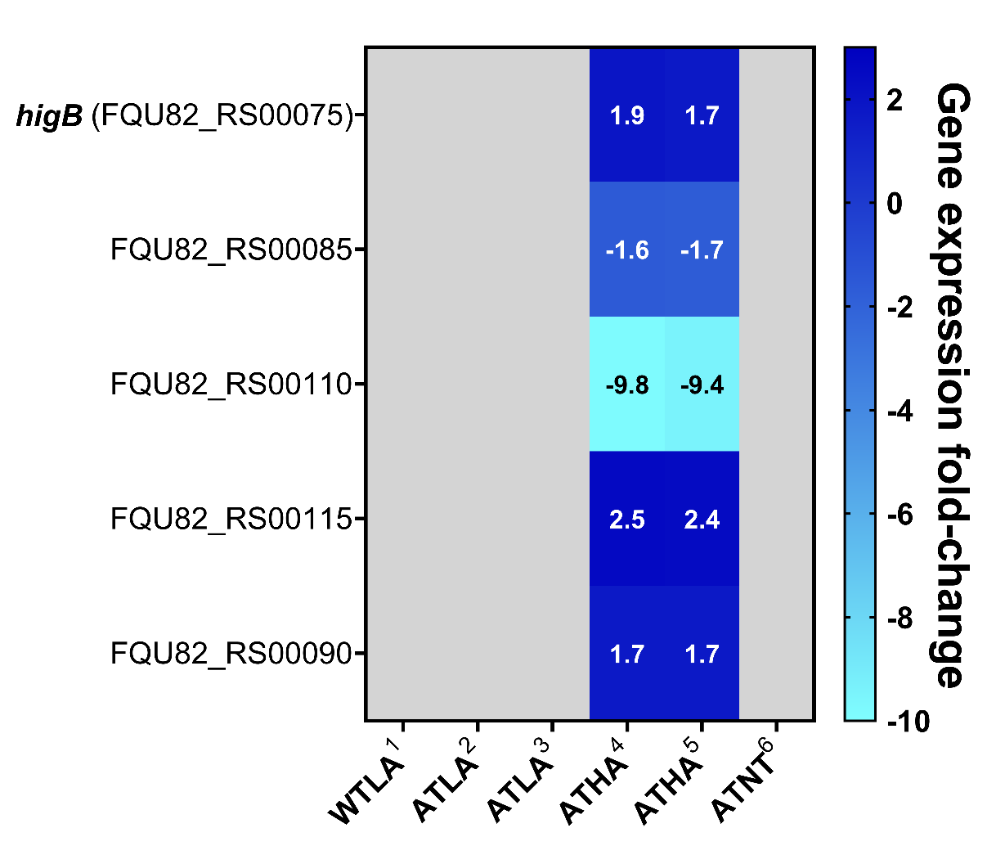


**B**

**Figure S10. Differentially expressed genes (DEGs) identified in plasmid p2 of WT, NAg^R^, and Ag^T^ *A. baumannii* ATCC 19606.** (**A**) Heat-map shows statistically significant (adj-*P* < 0.01) expression fold-change in plasmid-oriented genes in the WT and NAg^R^ strains. Pairwise comparison: ^1^ WTLN versus WTNT, ^2^ NRLN versus WTLN, ^3^ NRLN versus NRNT, ^4^ NRHN versus NRNT, ^5^ NRHN versus NRLN. (**B**) Statistically significant plasmid-oriented gene expression fold-changes in the WT and Ag^T^ strains. Pairwise comparisons: ^1^ WTLA versus WTNT, ^2^ ATLA versus WTLA, ^3^ ATLA versus ATNT, ^4^ ATHA versus ATNT, ^5^ ATHA versus ATLA, ^6^ ATNT vs WTNT. Genes with only locus tags provided are currently uncharacterised and have no known putative function. Fold-change values and scale (*right*) are included. Grey cells indicate statistically insignificant (adj-*P* > 0.01) fold-change for respective gene/mRNA transcript. DOI: 10.6084/m9.figshare.26011609
